# Integrating ultrasound radiomics and clinicopathological features for machine learning-based survival prediction in patients with nonmetastatic triple-negative breast cancer

**DOI:** 10.1186/s12885-025-13635-w

**Published:** 2025-02-18

**Authors:** Wenwen, Zekun Jiang, Jingyan Liu, Dingbang Liu, Yiyue Li, Yushuang He, Haina Zhao, Lin Ma, Yixin Zhu, Qiongxian Long, Jun Gao, Honghao Luo, Heng Jiang, Kang Li, Xiaorong Zhong, Yulan Peng

**Affiliations:** 1 Department of Ultrasound and West China Biomedical Big Data Center, West China Hospital, Sichuan University, No. 37 Guoxue Alley, Wuhou District, Chengdu, Sichuan 610000 China; 2 College of Computer Science, Sichuan University, Chengdu, Sichuan 610000 China; 3 West China School of Medicine, Sichuan University, Chengdu, Sichuan 610000 China; 4 Department of Ultrasonography, Peking University Shenzhen Hospital, Shenzhen, 515100 China; 5 Department of Pathology, The Affiliated Nanchong Central Hospital of North Sichuan Medical College, Nanchong, Sichuan 637000 China; 6 College of Medicine, University of Nebraska Medical Center, Omaha, NE 68198 USA; 7 Breast Disease Center, Cancer Center, West China Hospital, Sichuan University, Chengdu, Sichuan 610041 China; 8 Multi-omics Laboratory of Breast Diseases, State Key Laboratory of Biotherapy, Innovation Center for Biotherapy, West China Hospital, National Collaborative, Sichuan University, No. 37 Guoxue Alley, Wuhou District, Chengdu, Sichuan 610041 China; 9 Med-X Center for Informatics, Sichuan University, Chengdu, Sichuan 610041 China

**Keywords:** Triple negative breast cancer, Ultrasound, Radiomics, Prognosis, Machine learning

## Abstract

**Objective:**

This study aimed to evaluate the predictive value of implementing machine learning models based on ultrasound radiomics and clinicopathological features in the survival analysis of triple-negative breast cancer (TNBC) patients.

**Methods and materials:**

All patients, including retrospective cohort (training cohort, *n* = 306; internal validation cohort, *n* = 77) and prospective external validation cohort (*n* = 82), were diagnosed as locoregional TNBC and underwent pre-intervention sonographic evaluation in this multi-center study. A thorough chart review was conducted for each patient to collect clinicopathological and sonographic features, and ultrasound radiomics features were obtained by PyRadiomics. Deep learning algorithms were utilized to delineate ROIs on ultrasound images. Radiomics analysis pipeline modules were developed for analyzing features. Radiomic scores, clinical scores, and combined nomograms were analyzed to predict 2-year, 3-year, and 5-year overall survival (OS) and disease-free survival (DFS). Receiver operating characteristic (ROC) curves, calibration curves, and decision curves were used to evaluate the prediction performance.

**Findings:**

Both clinical and radiomic scores showed good performance for overall survival and disease-free survival prediction in internal (median AUC of 0.82 and 0.72 respectively) and external validation (median AUC of 0.70 and 0.74 respectively). The combined nomograms had AUCs of 0.80–0.93 and 0.73–0.89 in the internal and external validation, which had best predictive performance in all tasks (*p* < 0.05), especially for 5-year OS (*p* < 0.01). For the overall evaluation of six tasks, combined models obtained better performance than clinical and radiomic scores [AUCs of 0.83 (0.73,0.93), 0.81 (0.72,0.93), and 0.70 (0.61,0.85) respectively].

**Interpretation:**

The combined nomograms based on pre-intervention ultrasound radiomics and clinicopathological features demonstrated exemplary performance in survival analysis. The new models may allow us to non-invasively classify TNBC patients with various disease outcome.

**Supplementary Information:**

The online version contains supplementary material available at 10.1186/s12885-025-13635-w.

## Introduction

There were around 2.3 million newly diagnosed breast cancers in 2020 [1], which has become the most common worldwide and poses an increasing threat to women’s health. Triple-negative breast cancer (TNBC) is a subtype of breast cancer defined by the negative expression of estrogen receptors (ER), progesterone receptors (PR), and human epidermal growth factor receptor-2 (HER2), accounting for 15–20% of breast cancers [2]. For the more aggressive biology behavior, TNBC tends to have a poorer prognosis than other subtypes, with a higher risk of relapse, metastatic disease, and worse survival outcomes [2–5], and the median survival of TNBC patients with metastatic disease is around 13–18 months [6, 7]. Effective treatment of TNBC remains a clinical challenge due to its aggressive behavior and unfavorable outcome, thus, reflecting the vital role of accurate survival prediction and individualized management for these patients.

Ultrasound has been widely used in evaluating breast cancer with no radiation exposure and easy access in clinical practice [8, 9]. Many studies have been exploring its prognostic values for breast cancer. The breast lesions classified as BiRADS 4 A category in ultrasound might have a higher risk of recurrence than 4B-5 categories [10], which may be attributed to the association between benign-looking US features and delayed diagnosis and treatment. Wang et al. reported that the vertical orientation of the tumors on ultrasound images is correlated with worse recurrence-free survival and more axilla lymph node metastases for TNBC patients [11, 12]. Other studies showed that posterior acoustic enhancement, local skin edema, round or oval shape, and circumscribed margins were correlated with unfavorable prognostic outcomes [11–15].

However, interpretation of tumor images by radiologist mainly depends on semantic features but ignores many significant information derived from medical images. For the visual interpretation of the medical images, radiomics is an emerging discipline which aims to add further quantitative objectivity [16]. Radiomic features, a huge variety of mathematical descriptors, can be calculated from images quantifying different aspects of the tumor shape and texture. Radiomics investigates the ability of such features to characterize clinical properties of the lesions [17], and attempts to become a precious tool to support personalized clinical decisions [18]. Zheng et al. have established a predictive model for axilla lymph node metastasis by radiomics-based machine learning of preoperative ultrasound with an AUC value of 0.90 [19]. In the literature review, over 20 studies were found to have used artificial intelligence tools to predict the prognosis of TNBC (s-Table 1), proving that nomogram models or other AI tools could achieve good predictive performance. Of these, only five studies dealt with breast imaging examination (1 with mammography [20], 2 with MRI [21, 22] and 2 with ultrasound [23, 24]). Yu et al. proposed that ultrasound radiomics could be a potential biomarker to stratify TNBC patients into distinct prognostic groups and improve individualized management plans [23]. Wang et al. created a machine learning model based on ultrasound radiomics and clinicopathological features, which predicted disease-free survival (DFS) well in TNBC patients(AUC 0.84–0.90) [24].

There are limited resources on investigating the use of sonographic radiomics-based machine learning models in predicting TNBC patients’ long-term outcomes. And this study was designed to evaluate the prognostic predictive value of machine learning models based on clinicopathological features and ultrasound radiomics for disease outcomes in TNBC patients, further stratifying TNBC patients with different risk of prognosis.

## Methods

### Patient cohort

This cohort study was performed in West China Hospital of People’s Republic of China. Institutional Review Board approved this study (No. 2020 − 1219) and patients included in this study granted written permission for anonymized data use for research purposes at the time of biopsy or surgery.

**Fig. 1:**
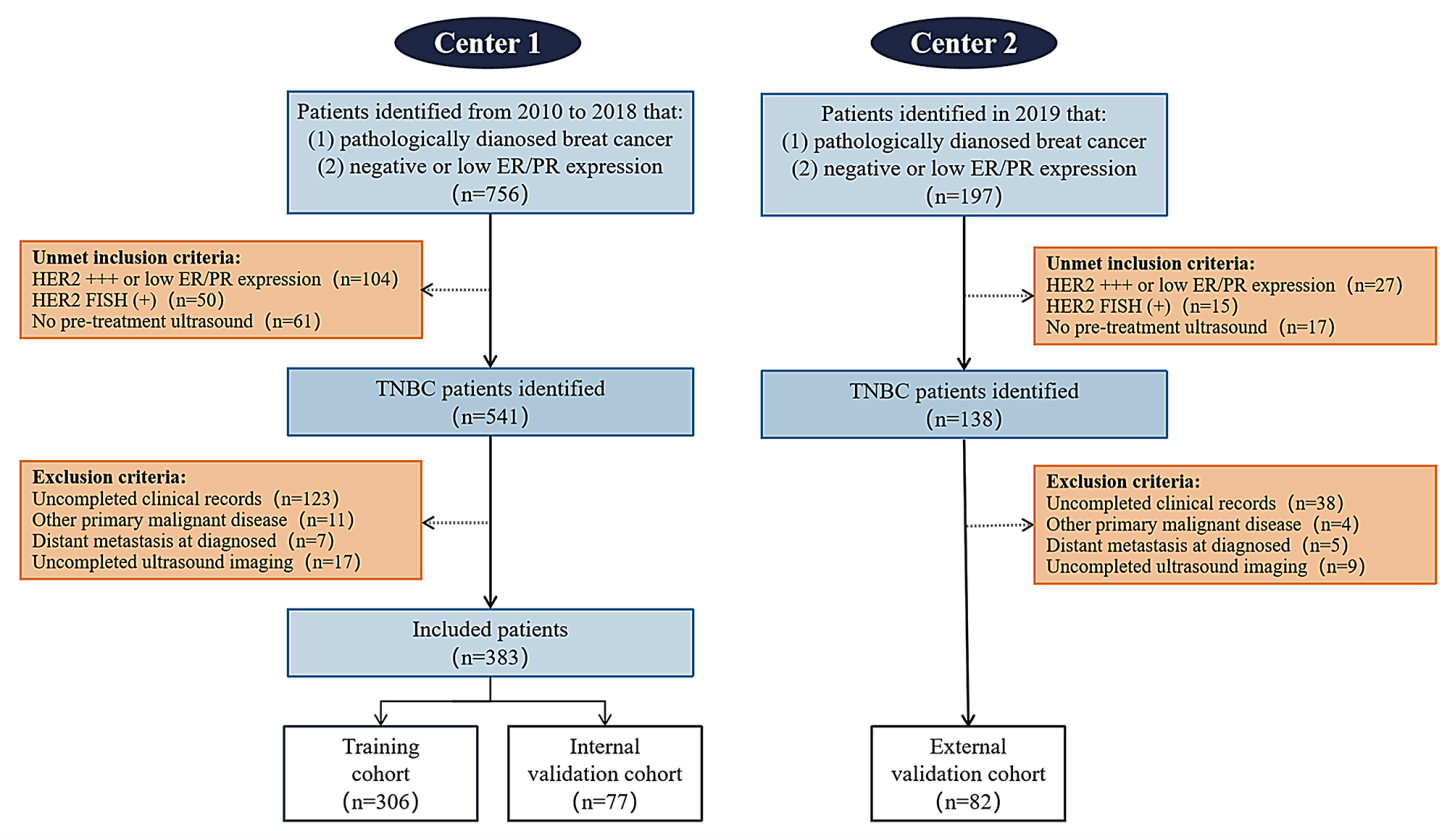
Patient selection flow diagram. Orange boxes represent patients that were excluded for the stated reason. TNBC, triple negative breast cancer; ER, estrogen receptor; PR, progesterone receptor; HER2, human epidermal growth factor receptor-2; FISH, fluorescence in situ hybridization

In this study, we included a retrospective cohort for training and internal validation from West China Hospital (center 1, Sichuan, China), and a prospective cohort for external validation from Nanchong Central Hospital (center 2, Sichuan, China). A total of 465 patients were enrolled (Fig. 1). For the retrospective cohort, women diagnosed with unilateral stage I to III TNBC and detailed pre-intervention ultrasound evaluation from 2010 to 2018 at center 1, were recruited. Inclusion criteria were: (i) pathologic diagnosis of triple-negative invasive ductal cancer; (ii) treated in our hospital; (iii) ultrasound examination performed before biopsy and within one month before intervention. Exclusion criteria were: (i) patients with distant metastatic; (ii) with primary malignancy of other sites; (iii) bilateral breast cancer; (iv) were pregnant or breastfeeding. The patients were prospectively followed through Breast Cancer Information Management System (BCIMS, see s-Method A) before December 2021 [25].

We grouped the center 1 data into a training cohort (*n* = 306) and an internal validation cohort (*n* = 77), which was no overlap in cases between the two datasets. In the same criteria, patients diagnosed at center 2 were prospectively enrolled in 2019 as independent external validation cohort (*n* = 82) and followed before February 2023.

Clinical profiles were recorded and derived from the BCIMS and electronic medical records. Pathologic types and receptor expressions of tumor were reviewed by the Department of Pathology at center 1 and 2 (s-Method B). Ultrasound was performed within 1 month before intervention (s-Method C). Ultrasound information of breast mass and lymph nodes, including major axis length, quadrant, number, axillary lymph node abnormality, clavicular lymph nodes abnormality, ipsilateral breast abnormality, contralateral breast abnormality, contralateral lymph nodes abnormality, were retrospectively collected from reports. Ultrasound images in retrospective cohort were derived from the Breast Ultrasound and Pathology Data Intelligent Management System of West China Hospital (s-Method D). One 2-dimension B-mode image of the tumors’ major axis planes was selected for radiomics analysis. Besides, selected images were independently reviewed by two senior radiologists specialized (Jingyan Liu and Yulan Peng) in breast blinded to survival outcomes, which BiRADS features and risk classification of lesions were evaluated and recorded. The height, width and height width ratio (HWR), obtained through a self-developed computer aided diagnosis tool, were used to describe orientation (s-Fig. 1). The vertical edge of the smallest parallel circumscribed rectangle of the tumor was recorded as height, the horizontal edge as width, and its ratio as HWR. Clinical, pathologic and sonographic features mentioned above and its origins were listed in s-Table 2. For the treatment of patients, treatment 1 was defined as surgery, treatment 2 as neoadjuvant chemotherapy followed by surgery, and treatment 3 as chemotherapy without surgery. The conduction of radiation therapy was all recorded. Other advanced therapy, including immunotherapy and proton therapy, were not recorded. The endpoints were overall survival (OS) and DFS. OS time is defined as the interval between the date of pathological diagnosis and death or last follow-up. DFS time were defined as the interval between the date of pathological diagnosis and progression of disease including metastasis, recurrence, or death of any reason.

### Tumor segmentation and feature extraction

**Fig. 2:**
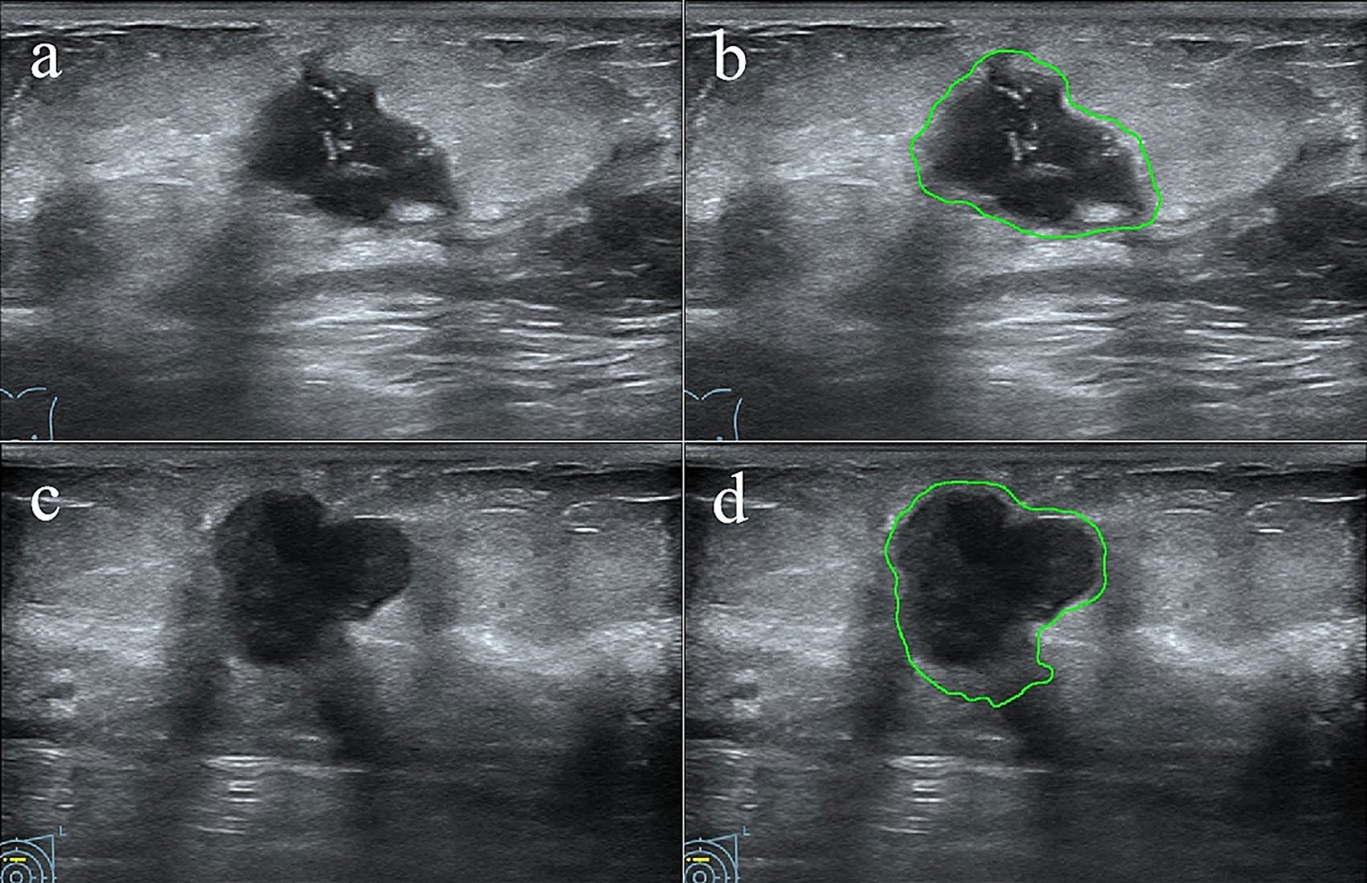
ROI segmentation examples by using YOLO V3

The overall machine learning workflow was shown in s-Fig. 2. A deep learning-based tumor segmentation model for breast ultrasound images was developed based on YOLO V3 algorithm [26] before (dice coefficient = 0.92), where the YOLO method has also been successfully applied to the segmentation of other tumors [26, 27]. Here, we used it to automatically segment the ROIs of ultrasound images (Fig. 2). Manual correction was performed by senior radiologist with more than 5 years of practice experience to obtain the accurate tumor boundaries.

Then, image preprocessing and radiomics feature extraction were implemented by using PyRadiomics library (version 3.0.1) [27, 28]. All ultrasound images were normalized and the grey level values were scaled by a factor of 100. Then, image discretization was performed with the binwidth of 5 and the voxel array shift of 300 to prevent negative values. In all, 103 quantified radiomics features were calculated including 12 shape-based features, 18 first-order features, and 73 texture features, which were described in detail in the PyRadiomics documentation (https://pyradiomics.readthedocs.io/en/latest/features.html). All feature classes were calculated on the preprocessed original image without any filtering and most features were consistent with feature definitions defined in the Imaging Biomarker Standardization Initiative (IBSI) [29].

### Radiomics analysis pipeline (RAP) module

**Fig. 3:**
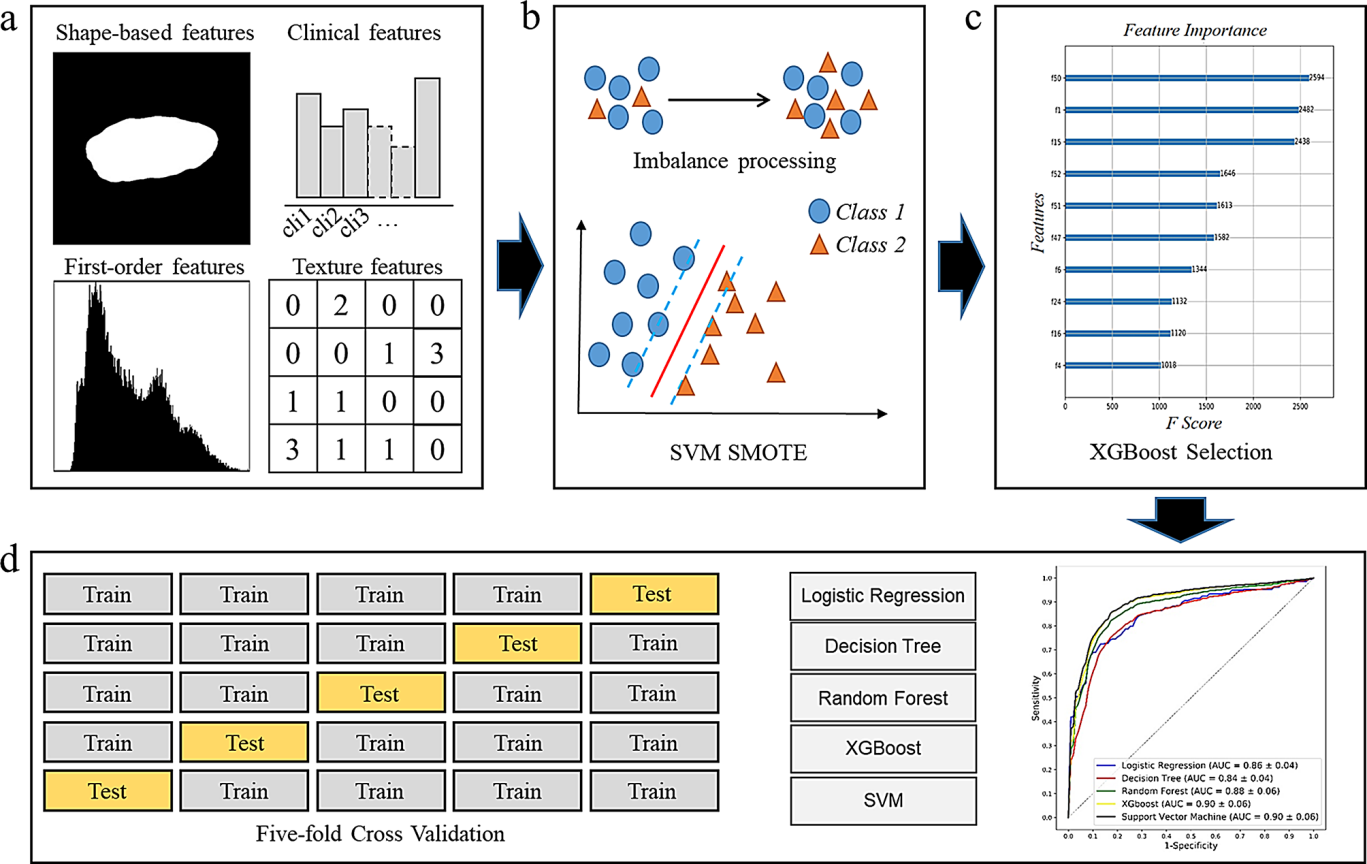
Radiomics analysis pipeline module

For processing the radiomic and clinical features efficiently and interpretably, a RAP module was designed. The details of RAP module were provided in Fig. 3. First, support vector machine (SVM) synthetic minority oversampling technique (SMOTE) was performed to solve the problem of data imbalance in the training cohort [30, 31]. The SVM SMOTE was a variant of SMOTE using SVM algorithm to detect samples for generating new synthetic samples, which was implemented by Imbalanced-learn [32] library (version 0.6.2). Then, based on the final training datasets, XGBoost [33, 34] was used to select the valuable features for survival prediction, with the max depth of 10 and lambda of 1, which was implemented by using xgboost library (version 0.82). Finally, the machine learning models were built and evaluated using 5-fold cross validation. Here, logistic regression, decision tree, random forest, XGBoost, and SVM algorithms were performed and compared using Scikit-learn library (version 0.24.2), respectively. The best modeling combination was used to build the final models.

### Radiomic scores, clinical scores, and combined nomograms

For predicting 2-year, 3-year, 5-year OS and DFS, radiomic scores and clinical scores were established respectively by inputting relevant features into the RAP module. The model scores, namely the prediction probability of the machine learning models, meant the probability of survival of the patient [35]. The higher the scores, the better the survival. Then, the combined nomograms were constructed by using the radiomic scores and clinical scores. The nomogram modeling was implemented by using “rms” package of R (version 3.5.3). For analyzing OS and DFS, radiomic scores and clinical scores were established respectively by inputting relevant features into the RAP module. All the machine learning models were built in the training cohort, then was tested in the internal and external validation cohorts. Due to the time constraints of the prospective collection, external validation was only assessed for 2-year and 3-year prognosis prediction.

### Performance assessment and statistical analysis

Statistical tests were performed using IBM SPSS Statistics (version 25.0), R (version 3.5.3) and Python (version 3.8.5). All tests were two-sided and P value < 0.05 was considered as statistically significant.

For the univariate analysis of clinicopathological and sonographic features among primary, internal validation and external validation cohort, categorical data were analyzed by Chi-square test, and continuous variables were analyzed by Mann-Whitney U or Kruskal-Wallis test. The ROC curves and calibration curves were used to evaluate the performance of machine learning models in all cohorts. DeLong test was used to compare the performance of different models. Decision curves [36] were performed to evaluate the clinical utility of nomograms in the validation cohort. The survival curves were drawn by using Kaplan-Meier method to demonstrate the clinical value of models.

Valuable features of all models were compared to address potential confounding bias, and model validation ensured against overfitting.

## Results

### Patient characteristics

A total of 465 patients were included in this study with a median follow-up time of 65.3 months, assigned into the training cohort (*n* = 306), internal validation cohort (*n* = 77), and external validation cohort (*n* = 82). The valuable clinicopathological and ultrasound characteristics of three cohorts are described in Table 1. All-cause mortality during the 3-year follow-up was 12.3% (*n* = 52), with 33(11.0%) patients from the training cohort, 9(16.7%) patients from internal validation cohort, and 10(14.5%) patients from the external validation cohort. 77 (18.2%) patients had disease progression [(training cohort, *n* = 53 (17.6%); internal validation cohort, *n* = 10 (18.5%); external validation cohort, *n* = 14(20.3%)] during the 3-year of follow-up, and the median DFS time was 52.3 months. Among patients with disease progression, 67 (14.6%) had distant metastases, and 10 (2.2%) presented local recurrence.

**Table 1:** Clinicopathological and sonographic features in the survival predictive models

Characteristics		Training (*N* = 306)	Internal validation (*N* = 77)	External validation (*N* = 82)	*P*-value
Age, median (range) (years)		49(28,81)	45(26, 81)	46.5(26, 81)	< 0.01
Symptom, n (%)	yes	56(18.30%)	16(20.78%)	22(26.83%)	0.23
	no	250(81.70%)	61(79.22%)	60(73.17%)	
Pathological T staging, n (%)	1	89(29.08%)	19(24.68%)	22(26.83%)	0.93
	2	185(60.46%)	48(62.34%)	48(58.54%)	
	3	15(4.90%)	4(5.19%)	5(6.10%)	
	4	17(5.56%)	6(7.79%)	7(8.54%)	
Pathological N staging, n (%)	0	166(54.25%)	31(40.26%)	36(43.90%)	0.14
	1	89(29.08%)	27(35.06%)	23(28.05%)	
	2	24(7.84%)	8(10.39%)	9(10.98%)	
	3	27(8.82%)	11(14.29%)	14(17.07%)	
Clinical staging	I	68(22.22%)	12(15.58%)	15(18.29%)	0.43
	II	172(56.21%)	44(57.14%)	43(52.44%)	
	III	66(21.57%)	21(27.27%)	24(29.27%)	
Treatment, n (%)	1	251(82.03%)	61(79.22%)	60(73.17%)	0.49
	2	44(14.38%)	12(15.58%)	17(20.73%)	
	3	11(3.59%)	4(5.19%)	5(6.10%)	
Mixed pathological types, n (%)	yes	104(33.99%)	19(24.68%)	22(26.83%)	0.19
	no	202(66.01%)	58(75.32%)	60(73.17%)	
HER-2 expression, n (%)	0	150(49.02%)	39(50.65%)	51(62.20%)	0.34
	1	99(32.35%)	24(31.17%)	20(24.39%)	
	2	57(18.63%)	14(18.18%)	11(13.41%)	
Ki-67 expression, median (range) (%)		60(5,95)	65(10, 95)	65(10, 95)	0.26
Tumor size, median (range) (mm)		24(6,65)	25(10, 65)	26(10, 65)	0.32
Quadrant, n (%)	upper outer	190(62.09%)	44(57.14%)	45(54.88%)	
	lower outer	36(11.76%)	11(14.29%)	11(13.41%)	
	lower inner	22(7.19%)	6(7.79%)	8(9.76%)	
	upper inner	58(18.95%)	16(20.78%)	18(21.95%)	
Posterior enhancement, n (%)		132(43.14%)	31(40.26%)	29(35.37%)	0.44
BI-RADS classification, n (%)	4b	29(9.48%)	8(10.39%)	9(10.98%)	0.99
	4c	124(40.52%)	30(38.96%)	33(40.24%)	
	5	153(50.00%)	39(50.65%)	40(48.78%)	
Height, median (range)		232(59, 505)	250(94, 488)	240.5(83,488)	0.16
Width, median (range)		342(107,763)	364(136,746)	355.5(136,763)	0.54
height width ratio, median(range)		0.69(0.29,1.34)	0.71(0.35,1.07)	0.73(0.36,1.30)	0.69
Clavicular lymph nodes, n (%)	yes	43(14.05%)	17(22.08%)	19(23.17%)	0.06
	no	263(85.95%)	60(77.92%)	63(76.83%)	
Contralateral breast abnormality, n (%)	yes	118(38.56%)	30(38.96%)	33(40.24%)	0.96
	no	188(61.44%)	47(61.04%)	49(59.76%)	

Clinicopathological and sonographic features attributed to the survival predictive models for training (*N* = 306), internal validation (*N* = 77) and external validation cohort (*N* = 82) are displayed as median (range) for continuous variables and count (percent) for categorical variables. Age is represented in years, and tumor size are presented by major axis of lesions (mm)

### Radiomics scores, clinical scores and valuable features

After RAP processing, different machine learning models based on radiomic and clinical features were built respectively by using 5-fold cross validation in the training cohort. The radiomic and clinical scores were built using valuable features (s-Table 3), which were selected by XGBoost algorithm. XGBoost assigned features importance by evaluating the contribution of features in building gradient boosting trees, and feature selection was performed in order of importance. For clinical scores, age, tumor size, pathological N staging, height, width and HWR were taken as contributing factors in six tasks. For radiomic scores, the contribution ratio of the shape-based, first-order, and texture features was 5:10:16, and only the 10 Percentile of first-order feature was the common feature.

**Fig. 4:**
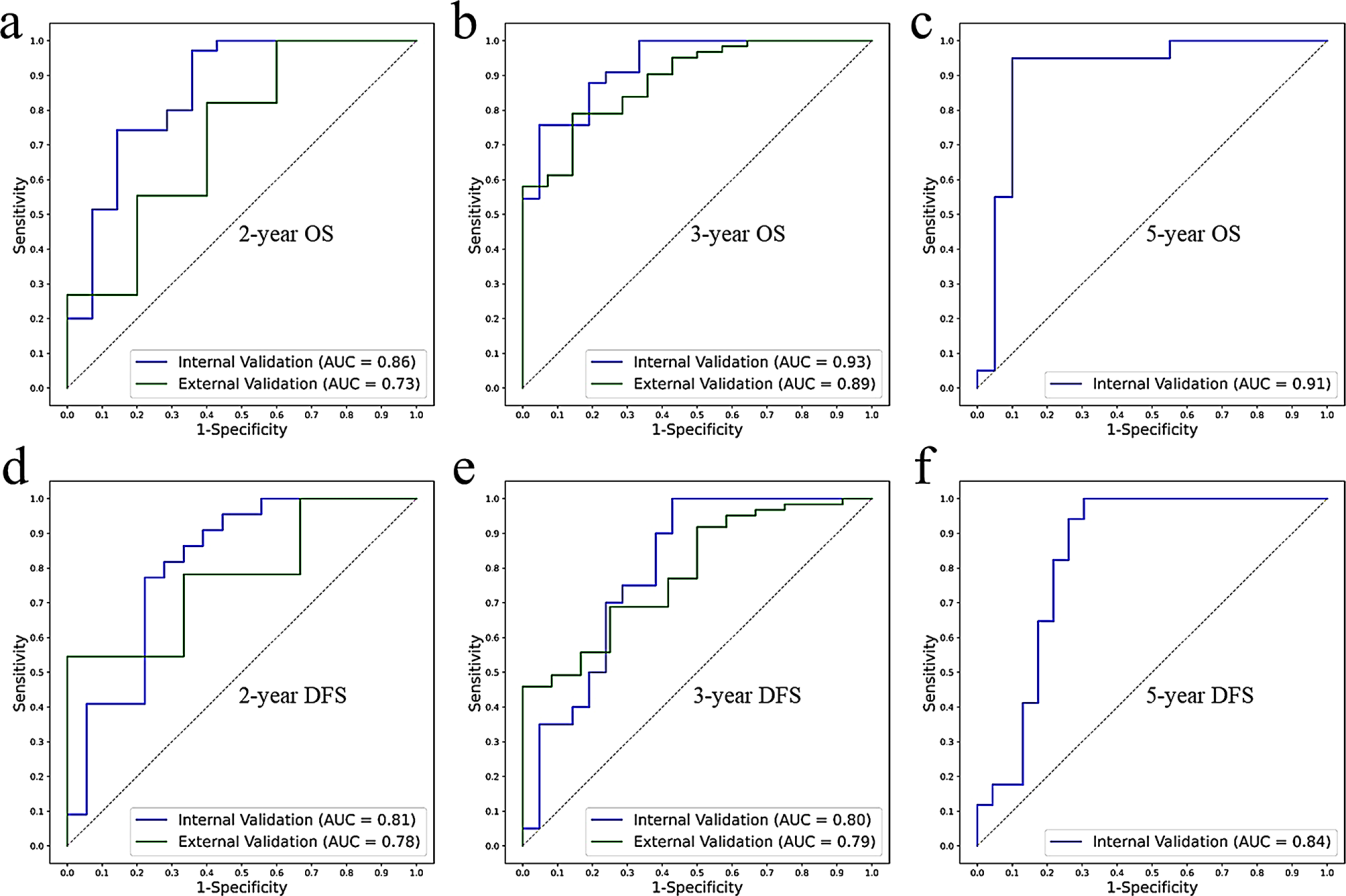
The ROC curves of combined nomograms in internal and external validation

The ROC curves of training cohort were conducted in different radiomic and clinical models (s-Fig. 3). SVM algorithm all achieved the optimal results on OS and DFS prediction. The prediction performance of clinical and radiomic scores were evaluated in internal and external validation cohort (s-Fig. 4). In the internal validation cohort, AUCs of all the scores are 0.65–0.93. As shown in Table 2, the scores for 2-year, 3-year OS/DFS also had AUCs of 0.61–0.93 in external validation.

**Table 2:** The prediction performance of radiomic scores, clinical scores, and combined nomograms in internal and external validation. OS, overall survival; DFS, disease-free survival

Tasks	Models	Internal validation					External validation				
		AUC	ACC	Precision	Recall	F1	AUC	ACC	Precision	Recall	F1
2-year OS	Clinical	0.83	0.88	0.85	1.00	0.92	0.72	0.90	0.93	0.96	0.95
	Radiomic	0.68	0.78	0.79	0.93	0.85	0.71	0.90	0.96	0.93	0.94
	**Combined**	**0.86**	**0.88**	**0.87**	**0.97**	**0.92**	**0.73**	**0.92**	**0.95**	**0.96**	**0.96**
3-year OS	Clinical	0.92	0.85	0.81	0.96	0.88	0.93	0.91	0.96	0.93	0.95
	Radiomic	0.77	0.71	0.69	0.82	0.75	0.70	0.79	0.94	0.81	0.87
	**Combined**	**0.93**	**0.87**	**0.83**	**1.00**	**0.90**	**0.89**	**0.86**	**0.92**	**0.90**	**0.91**
5-year OS	Clinical	0.84	0.80	0.71	1.00	0.83	/	/	/	/	/
	Radiomic	0.85	0.70	0.65	0.85	0.74	/	/	/	/	/
	**Combined**	**0.91**	**0.83**	**0.76**	**0.95**	**0.84**	/	/	/	/	/
2-year DFS	Clinical	0.80	0.70	0.69	0.82	0.75	0.75	0.81	0.98	0.82	0.89
	Radiomic	0.73	0.75	0.73	0.86	0.79	0.70	0.83	0.96	0.86	0.90
	**Combined**	**0.81**	**0.75**	**0.73**	**0.86**	**0.79**	**0.78**	**0.83**	**0.96**	**0.86**	**0.90**
3-year DFS	Clinical	0.77	0.71	0.65	0.85	0.74	0.72	0.77	0.89	0.82	0.86
	Radiomic	0.65	0.59	0.55	0.80	0.65	0.61	0.78	0.87	0.87	0.87
	**Combined**	**0.80**	**0.76**	**0.68**	**0.95**	**0.79**	**0.79**	**0.85**	**0.89**	**0.93**	**0.91**
5-year DFS	Clinical	0.81	0.73	0.64	0.82	0.72	/	/	/	/	/
	Radiomic	0.70	0.70	0.60	0.88	0.71	/	/	/	/	/
	**Combined**	**0.84**	**0.80**	**0.71**	**0.88**	**0.79**	/	/	/	/	/

### Combined nomograms

**Fig. 5:**
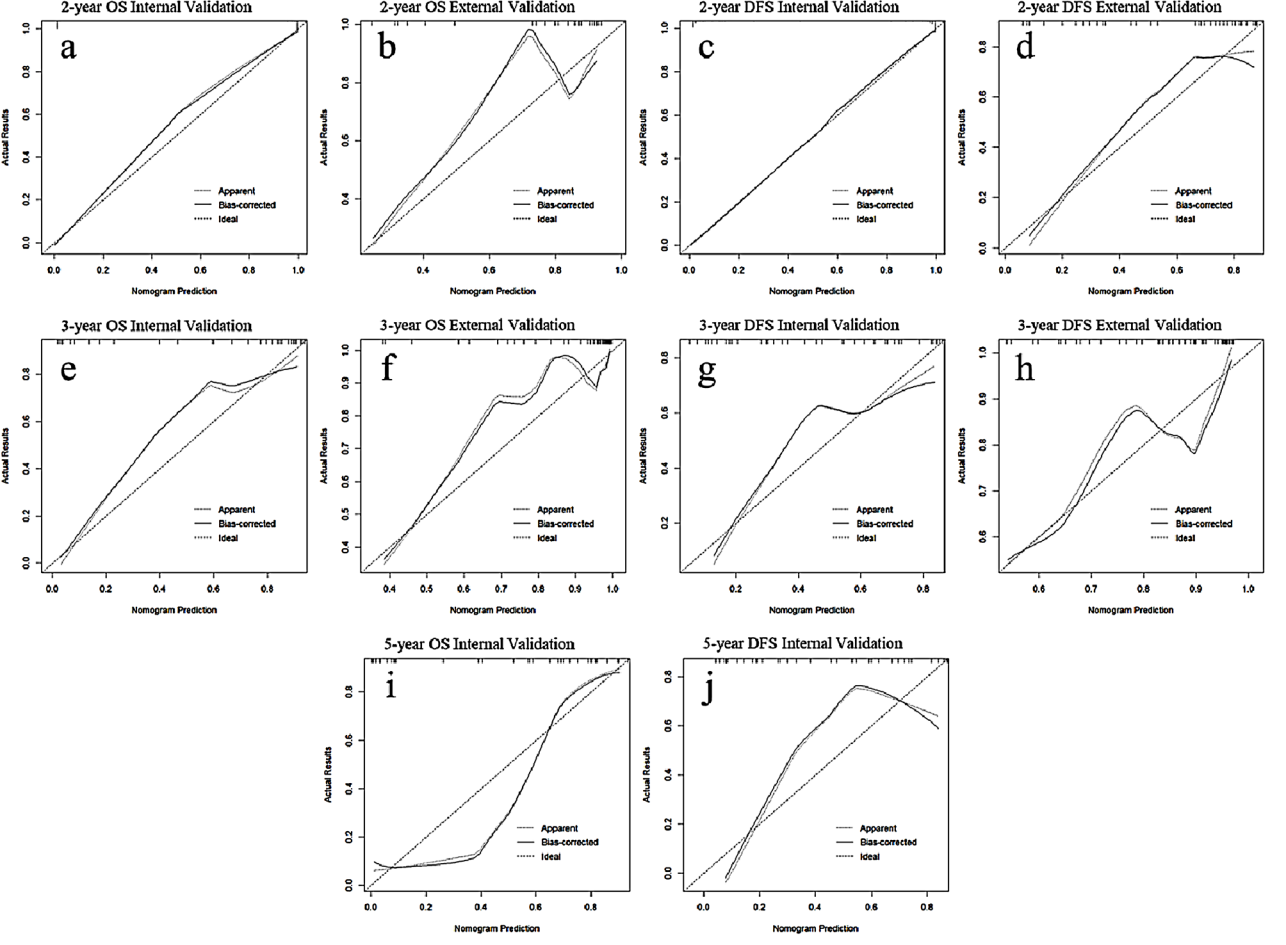
The calibration curves of combined nomograms in internal and external validation

The combined nomograms were established by using radiomic and clinical scores (s-Fig. 5). The performance comparison of all the radiomic scores, clinical scores, and combined nomograms could be seen in Table 2. In the internal validation cohort, the combined nomograms performed well in all tasks, with AUC of 0.80–0.93, ACC of 0.75–0.88, precision of 0.68–0.87, recall of 0.86-1.00, and F1 of 0.72–0.92 for all models (Fig. 4; Table 2). The combined model had best predictive performance in all internal validation tasks (*p* < 0.05), especially for 5-year OS (*p* < 0.01). In the external validation cohort, the combined nomograms had AUC of 0.73–0.89, ACC of 0.83–0.92, precision of 0.89–0.96, recall of 0.90–0.96, and F1 of 0.86–0.95. The combined models also got higher efficiency among three models in external validation (*p* < 0.05), except for 3-year OS (AUC of 0.93, 0.70 and 0.89 for clinical, radiomic and combined scores respectively).

The subgroup analysis of predictive performance of all models were listed in Table 3. For the efficiency evaluation of all tasks, combined models obtained better performance than clinical scores [AUC of 0.83(0.73,0.93) and 0.81(0.72,0.93) respectively] and radiomic scores [AUC of 0.70(0.61,0.85)]. Comparing to DFS, the models showed significantly better performance in OS prediction, (median AUC of 0.84 and 0.77). When it comes to different period of follow-up, the best performance of models was achieved in 5-year group (median AUC of 0.74, 0.78 and 0.84 for 2-year, 3-year and 5-year respectively).

**Table 3:** Subgroup analysis of prediction performance in internal and external validation. OS, overall survival; DFS, disease-free survival

Subgroup	Models	AUC Median(range)	ACC Median(range)	Precision Median(range)	Recall Median(range)	F1 Median(range)
Overall		0.79(0.61,0.93)	0.80(0.59,0.92)	0.82(0.55,0.98)	0.88(0.80,1.00)	0.87(0.65,0.96)
Outcome	OS	**0.84(0.68**,**0.93)**	**0.86(0.70**,**0.92)**	**0.85(0.65**,**0.96)**	**0.95(0.81**,**1.00)**	**0.90(0.74**,**0.96)**
	DFS	0.77(0.61,0.84)	0.76(0.59,0.85)	0.73(0.55,0.98)	0.86(0.80,0.95)	0.79(0.65,0.91)
Follow-up time	2-year	0.74(0.68,0.86)	**0.83(0.70**,**0.92)**	**0.90(0.69**,**0.98)**	**0.90(0.82**,**1.00)**	**0.90(0.75**,**0.96)**
	3-year	0.78(0.61,0.93)	0.79(0.59,0.91)	0.85(0.55,0.96)	0.89(0.80,1.00)	0.87(0.65,0.95)
	5-year	**0.84(0.70**,**0.91)**	0.77(0.70,0.83)	0.68(0.60,0.76)	0.88(0.82,1.00)	0.77(0.71,0.84)
Feature	Clinical	0.81(0.72,0.93)	0.81(0.70,0.91)	0.83(0.64,0.98)	0.89(0.82,1.00)	0.87(0.72,0.95)
	Radiomic	0.70(0.61,0.85)	0.77(0.59,0.90)	0.76(0.55,0.96)	0.86(0.80,0.93)	0.82(0.65,0.94)
	Combined	**0.83(0.73**,**0.93)**	**0.84(0.75**,**0.92)**	**0.85(0.68**,**0.96)**	**0.94(0.86**,**1.00)**	**0.90(0.79**,**0.96)**

### Clinical utility

**Fig. 6:**
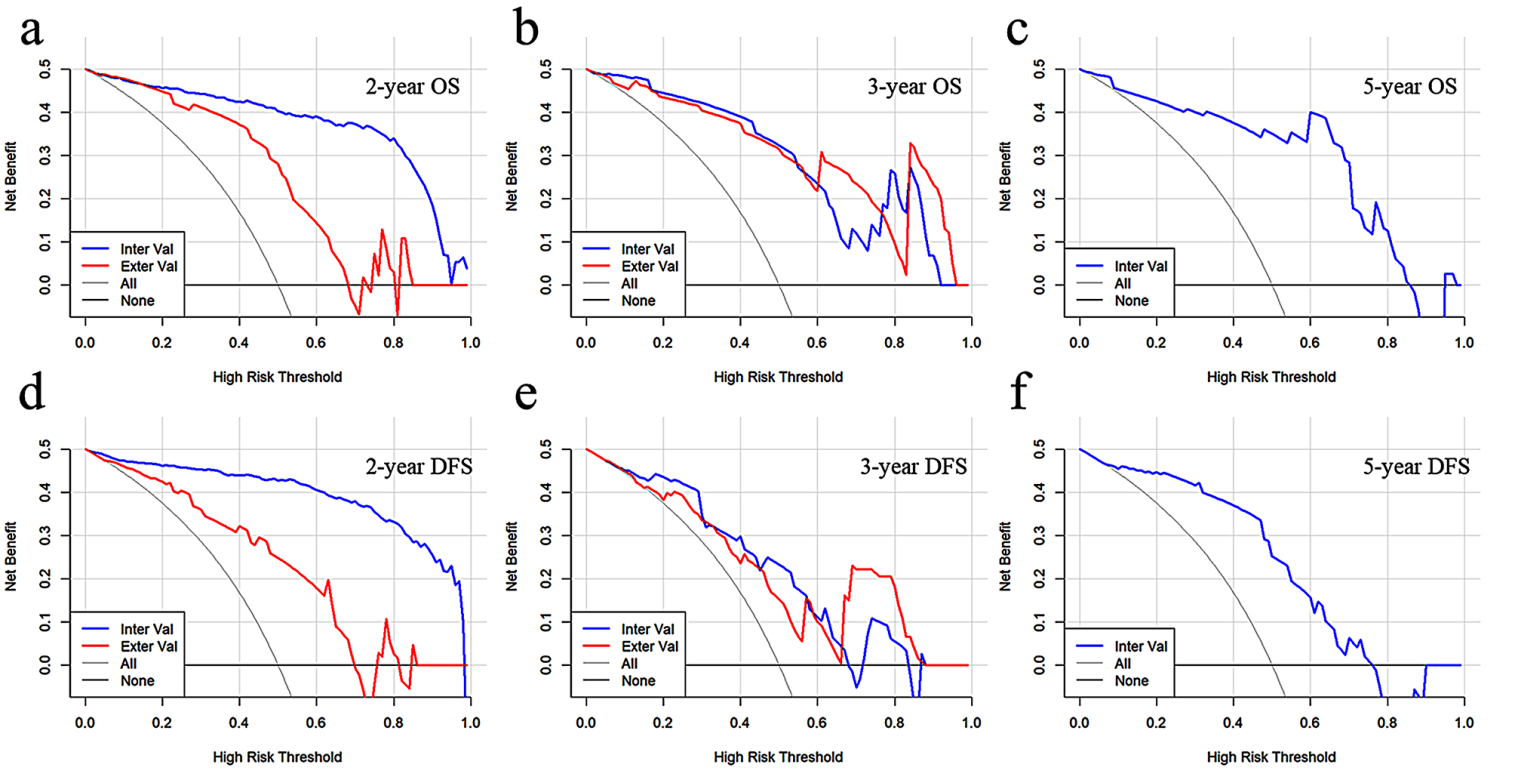
The decision curves of combined nomograms in internal and external validation

For further confirming the clinical gain of the final models, the calibration curves and decision-curve analysis was implemented (Figs. 5 and 6). Using nomograms for survival prediction all added more clinical benefit. The 3-year OS nomogram could add more benefit than using the treat-all scheme or the treat-none scheme at any given threshold of probability in all cohorts.

**Fig. 7:**
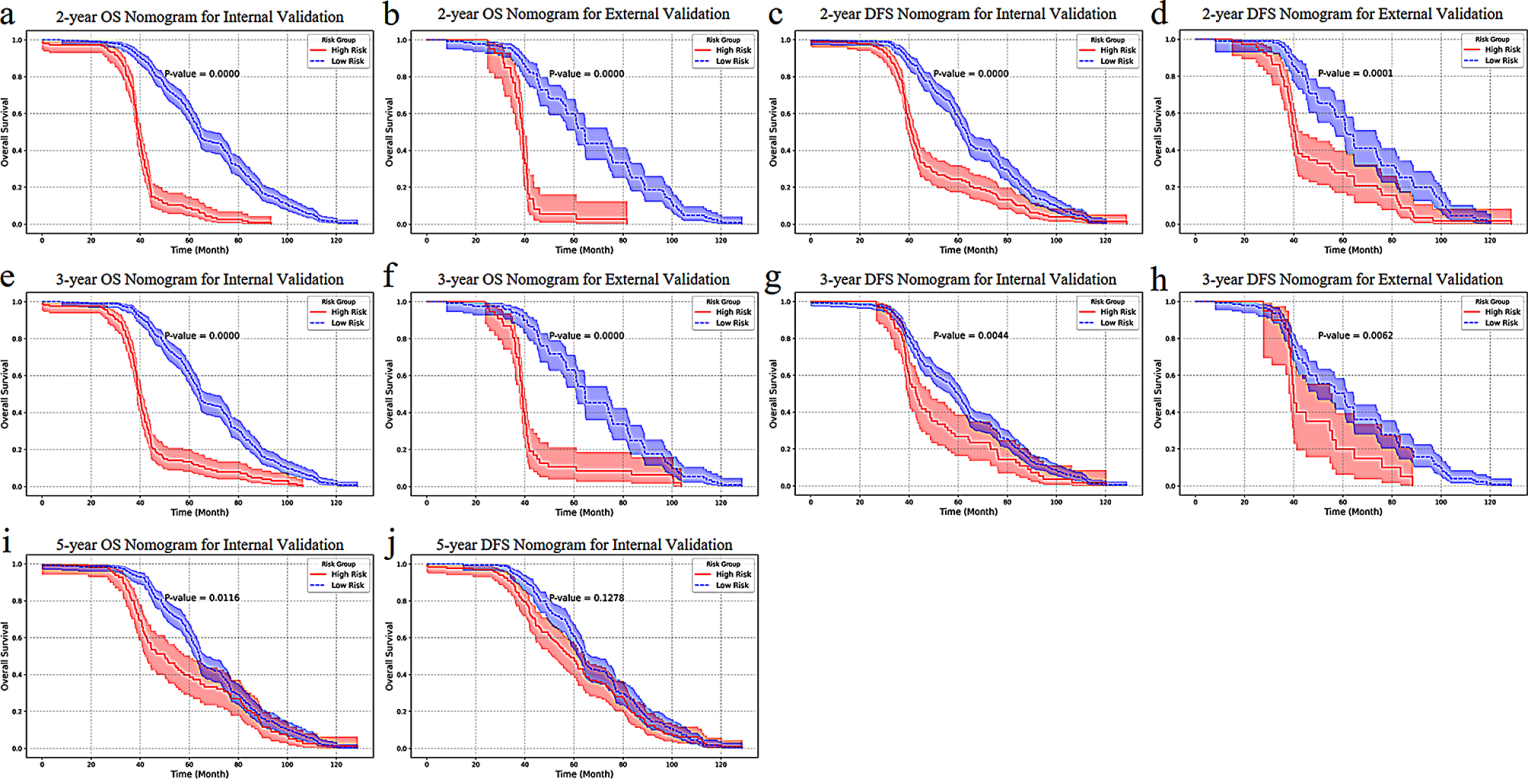
The Kaplan-Meier curves of all models in internal and external validation

According to the 2-year DFS, the patients were divided into low-risk group and high-risk group in tow hospitals (s-Table 4). In center 1, lesion palpability, clinical staging, neoadjuvant chemotherapy, and spiculate margin are independent risk factors for 2-year DFS (*p* = 0.002, 0.041, 0.017, 0.009 respectively). In center 2, age, lesion palpability, surgical history, and height are independent risk factors (*p* = 0.002, 0.017, 0.028, 0.026). All models have a certain potential for risk stratification of patients according to the prediction probability of clinical score, radiomic score and combined nomogram (s-Table 5). As illustrated by the Kaplan-Meier curves (Fig. 7), the combined nomograms for 2-year OS and 2-year DFS demonstrated superior performance in stratifying patients with varying prognostic risks (*p* < 0.05).

## Discussion

Understanding prognosis is critical for clinical decision-making and risk stratification of cancer patients. In this study, we developed and validated clinical scores, radiomic scores based on pre-treatment ultrasound and combined nomograms by machine learning to predict the survival outcomes of locoregional TNBC patients. Combined nomograms had significantly improved performance in different evaluation indexes compared to clinical scores, which means that ultrasound radiomics can improve the prognosis prediction efficiency of only using clinicopathological features. In other words, ultrasound radiomics and clinicopathological features are complementary for the prognosis prediction, rather than redundant information. Performance assessment proved that combined nomograms have generally better prediction performance (*P* < 0.05), which indicated the combination of radiomic and clinicopathological features was helpful in predicting the prognosis of cancer diseases.

In literature review, 21 articles were found using AI tools to predict the prognosis of TNBC (s-Table 1). The existing studies mainly focus on clinical, pathological, experimental, and radiological features, and reported varied prediction performance. In all listed studies, 8 studies only included clinicopathological features, 8 studies take experimental biomarkers as risk factors, and only 5 studies ensembled radiological features to predict disease outcomes of patients. The best performance model of studies using clinicopathological factors to predict prognosis achieve AUCs of 0.84–0.91 [37], similar to our clinical scores (AUC 0.72–0.93). Besides, Yu et al. [23] and Wang et al. [24] used ultrasound radiomic features to predict DFS of TNBC patients, achieving a C-index of 0.75 and AUC of 0.84–0.90 respectively, higher than ours. Breast radiomics have also been used with MRI and mammography to predict DFS and systemic recurrence with C-index of 0.87 [21] and 0.94 [20] and AUC of 0.93 [22]. Compared to reported radiomics-based models, our combined nomograms showed good performance in OS prediction, especially for 3-year OS. Our results offer some preliminary evidence of improvement in performance with combined nomograms. Besides, the results were promising, especially for the external validation, demonstrating the models’ robustness and generalizability. Our models were developed using existed clinical features, including clinicopathological data and ultrasound images. Consequently, the combined nomograms can offer predictive prognostic assessments at pre-interventional stage without necessitating additional procedures, thereby potentially enhancing clinical decision-making.

In the process of clinical scores building, clinicopathological and sonographic features was analyzed for prognosis prediction. Several features, including age, tumor size, pathological N staging, height, width and HWR were found contributing to all endpoints. Consistently with previous research, age, tumor size and pathological N staging are important risk factors of TNBC prognosis, showing highly correlation to OS and DFS in this study. Orientation of tumor has been reported to be risk factors of TNBC prognosis in existing studies, and tumors with major axis vertical to skin tends to have poor prognosis, which is similar to our results [38, 39]. Besides, Li et al. found that quadrant of tumors is related to prognosis of TNBC patients, demonstrating that tumor in the inner quadrant showed more metastases and recurrence than other quadrant [40].

We found that shape-based, first-order, and texture features all have contributions to the construction of machine learning models, indicating the potential value of ultrasound radiomic features in predicting the survival prognosis of breast cancer. In the radiomic scores, 10Percentile was the only common feature. The first-order features reflect the distribution of voxel intensities within the tumor region in the ultrasound image, where 10Percentile means the 10th percentile of a set of N_p_ voxels included in the ROI. However, it was extensively observed that the use of different parameters during acquisition and post-acquisition may modify the image signal with a significant impact on the value of the radiomic features, even when the diagnostic quality of the image is maintained [41, 42]. Radiomics can be applied successfully in the clinical practice only if the radiomic-based predictive models are robust and generalizable, which currently present significant challenges. These radiomic features may be further analyzed and validated in a larger cohort study in the future.

RAP module could be used as a plug-and-play tool for the diagnosis studies of various cancer diseases. The imbalance processing could effectively solve the problem of data imbalance in binary classification. Several studies have shown that SMOTE and its derivative algorithms can significantly improve the performance of machine learning models [43, 44]. In structured data processing, XGBoost had always demonstrated excellent processing ability and could select the valuable features from heterogeneous data [43, 45, 46]. Finally, the cross-validation and multiple machine learning modeling methods could determine the most accurate and robust prediction model.

Radiomics quality score (RQS) was a strong tool to validate the overall quality of radiomics study [47]. The RQS analysis of our study is 30 (s-Table 6). Compared with previous studies, the research quality was better [48, 49]. RQS self-examination should be a necessary part of radiomics research, which indicates whether there are any quality improvement details in this study. In a large investigate study in oncologic radiomics [48], the mean RQS score in 77 radiomics articles was only 9.4 and the highest was 21. Our RQS score was 30, preliminarily showing a good research quality.

There are some limitations to our radiomics study. First, this is a multi-center analysis consisted of retrospective and prospective cohorts, while the prospective cohort was only followed for 3 years. Generalizability can be improved if data from additional institutions and long-term follow-up is added. Second, we only chose the major axis planes each lesion to conduct the ultrasound radiomics. This major axis planes was selected according to BI-RADS [50] demonstrating that it is the most representative scene of breast mass and where to mainly interpret imaging features. As a lesion is a three-dimensional object, radiomics on different planes of mass could obviously offer more comprehensive information. Our findings open the possibility for future studied to shed light on the value of other ultrasound modalities and machine learning assessment of TNBC prognosis.

In Conclusion, ultrasound radiomics can improve the prognosis prediction efficiency of clinicopathological features in TNBC. The combined machine learning models showed good performance in predicting OS and DFS, which can help us non-invasively identify which patients are at high risk of poor prognosis and generate individualized clinical management. In future studies, enhancing the generalization performance of the model and investigating its practical clinical transformation will constitute a critical area in subsequent artificial intelligence research.

## Electronic supplementary material

Below is the link to the electronic supplementary material.

Supplementary Material 1

## Data Availability

Due to the privacy of patients, the original data are not available for public access but can be obtained from Yulan Peng (yulanpeng520@126.com) upon reasonable request. The main codes and examples are available in a publicly accessible repository (https://github.com/JZK00/radiomics_survival_analysis/tree/main/USRadiomics).
