# Supplementary material for: Integrating ultrasound radiomics and clinicopathological features for machine learning-based survival prediction in patients with nonmetastatic triple-negative breast cancer
Source: BMC Cancer. 2025 Feb 18;25:291. doi: 10.1186/s12885-025-13635-w (PMC11837701; doi:10.1186/s12885-025-13635-w)
Supplement: Supplementary file 1 — Supplementary Material 1 [file 12885_2025_13635_MOESM1_ESM.docx]

**Supplementary materials**

Contents

[s-Methods 3](#_Toc188393887)

[A. Breast Cancer Information Management System and follow-up 3](#_Toc188393888)

[B. Pathologic evaluation 3](#_Toc188393889)

[C. Details of ultrasound image acquisition. 3](#_Toc188393890)

[D. Breast Ultrasound and Pathology Data Intelligent Management System 3](#_Toc188393891)

[s-Figures 5](#_Toc188393892)

[s-Figure 1 Definition of height, width, and height width ratio. 5](#_Toc188393893)

[s-Figure 2 Ultrasound radiomics-based machine learning workflow 6](#_Toc188393894)

[s-Figure 3. The ROC curves of different machine learning models in the training cohort. 7](#_Toc188393895)

[s-Figure 4. The ROC curves of clinical and radiomic scores in internal and external validation. 8](#_Toc188393896)

[s-Figure 5. The combined nomograms. 9](#_Toc188393897)

[s-Tables 10](#_Toc188393898)

[s-Table 1. Literature review summary. 10](#_Toc188393899)

[s-Table 2 Patient characteristic of training, internal validation and external validation cohorts. 13](#_Toc188393900)

[s-Table 3 Valuable features across different radiomic and clinical scores. 18](#_Toc188393901)

[s-Table 4. Comparison of clinicopathological and sonographic characteristics between progressed and non-progressed patients in two center. 20](#_Toc188393902)

[s-Table 5 Survival analysis of prediction probability of clinical score, radiomic score and combined nomograms. 24](#_Toc188393903)

[s-Table 6. RQS^[22]^ analysis of the study. RQS, radiomics quality score. 25](#_Toc188393904)

[Reference 27](#_Toc188393905)

s-Methods

## **Breast Cancer Information Management System and follow-up**

Breast Cancer Information Management System (BCIMS) is a breast cancer database of Chinese breast cancer patients, including over 16,000 patients. Follow-up schedule was initially every 3 months for the first 2 years after diagnosis, then every 6 months from 3 to 5 years, and every year thereafter. Follow-up was conducted via outpatient visits, or by telephone or postal contact by research assistants.

## **Pathologic evaluation**

A nuclear staining <1% tumor specimen was defined as ER or PR negativity. The positive expression of HER2 was determined by immunohistochemistry (IHC) 3+ or positive on fluorescence in situ hybridization (FISH), while others are negative. Other pathologic data including mixed pathological types, histologic grade, Ki67 expression and lymph node involvement were also recorded.

## Details of ultrasound image acquisition.

Ultrasound images were acquired using machines equipped with a linear array high-frequency probe with a frequency range of 4 to 15 MHz, including the Philips (HD11, IU22, EPIQ5; Philips, Amsterdam, Netherlands), GE Logiq E9 (GE Healthcare, Chicago, IL, USA), HI VISION Preirus (Hitachi Medical Corp., Tokyo, Japan), Esaote MyLab90 (Esaote, Maastricht, Holland), Supersonic Imagine (Aix-en-Province, France), and Siemens ACUSON OXANA 2 (Siemens Healthineers, Erlangen, Germany). Ultrasound examination used a portable probe for radial scanning of the whole breast. The standard acquisition procedure for ultrasound images is to obtain longitudinal and transverse sections at the maximum diameter of the lesion with the lesion located in the middle of the image. Supplementary images are obtained that show any malignant signs of the lesion, such as calcification or structural distortion.

## **Breast Ultrasound and Pathology Data Intelligent Management System**

Breast Ultrasound and Pathology Data Intelligent Management System (BUPDIMS) contains electronic records of breast ultrasound examinations dating back to 2004 and electronic records of pathology examinations starting in 2009, including ultrasound reports, ultrasound images and pathology reports. Patients with tumor size over 65mm or lesions cannot be completely presented on the image was excluded.

s-Figures

## s-Figure 1 Definition of height, width, and height width ratio.

The vertical edge of the smallest parallel circumscribed rectangle of the tumor was recorded as height, the horizontal edge as width, and its ratio as HWR. The height width ratio (HWR) is used to describe orientation, which is defined as the ratio of width to length of the smallest rectangle connected to the tumor parallel to the coordinate system. The vertical edge of the smallest parallel circumscribed rectangle of the tumor was recorded as height, the horizontal edge as width, and its ratio as HWR.


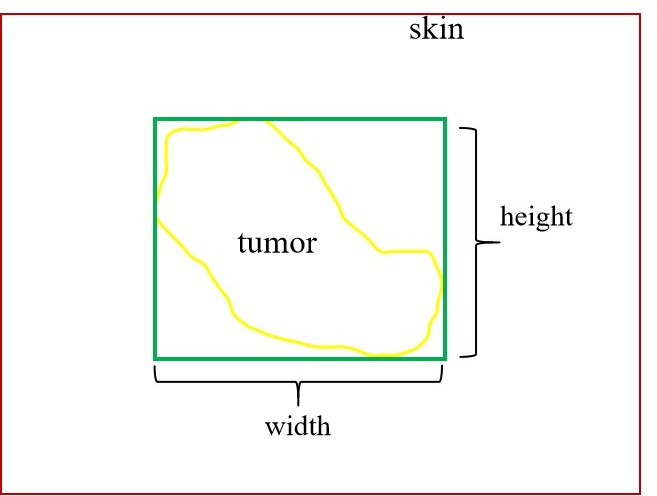


## **s-Figure 2 Ultrasound radiomics-based machine learning workflow**

The ultrasound images with ROIs and clinical characteristics were analyzed by using a RAP module in the training cohort. Then, 6 radiomic scores, clinical scores, and combined nomograms for 2-year, 3-year, 5-year OS and DFS were established, respectively. All the selected features and models were evaluated in the validation cohorts. US, ultrasound; ROI, region of interests; RAP, radiomics analysis pipeline; OS, overall survival; DFS, disease-free survival.


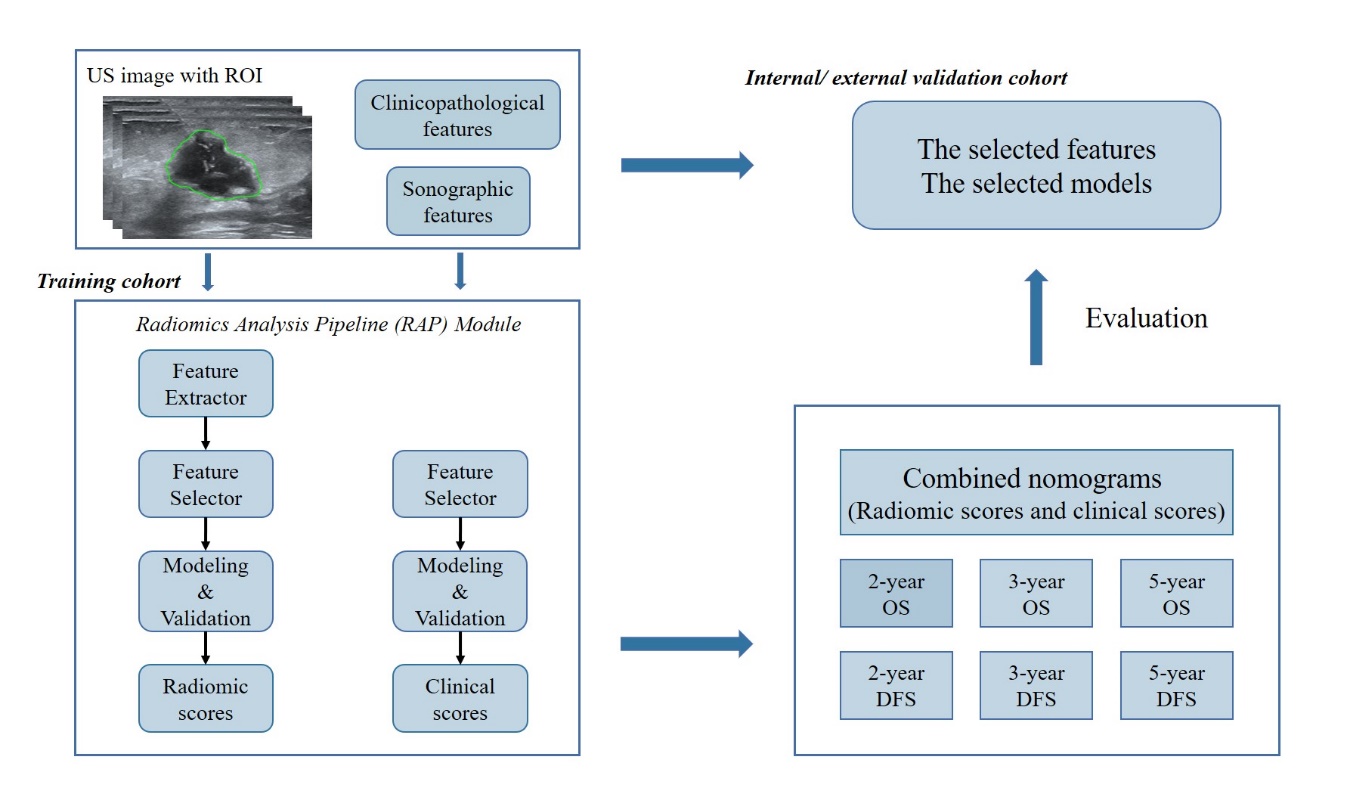


## **s-Figure 3. The ROC curves of different machine learning models in the training cohort.**

OS, overall survival; DFS, disease free survival; ROC, receiver operating characteristic; AUC, area under the ROC curve.


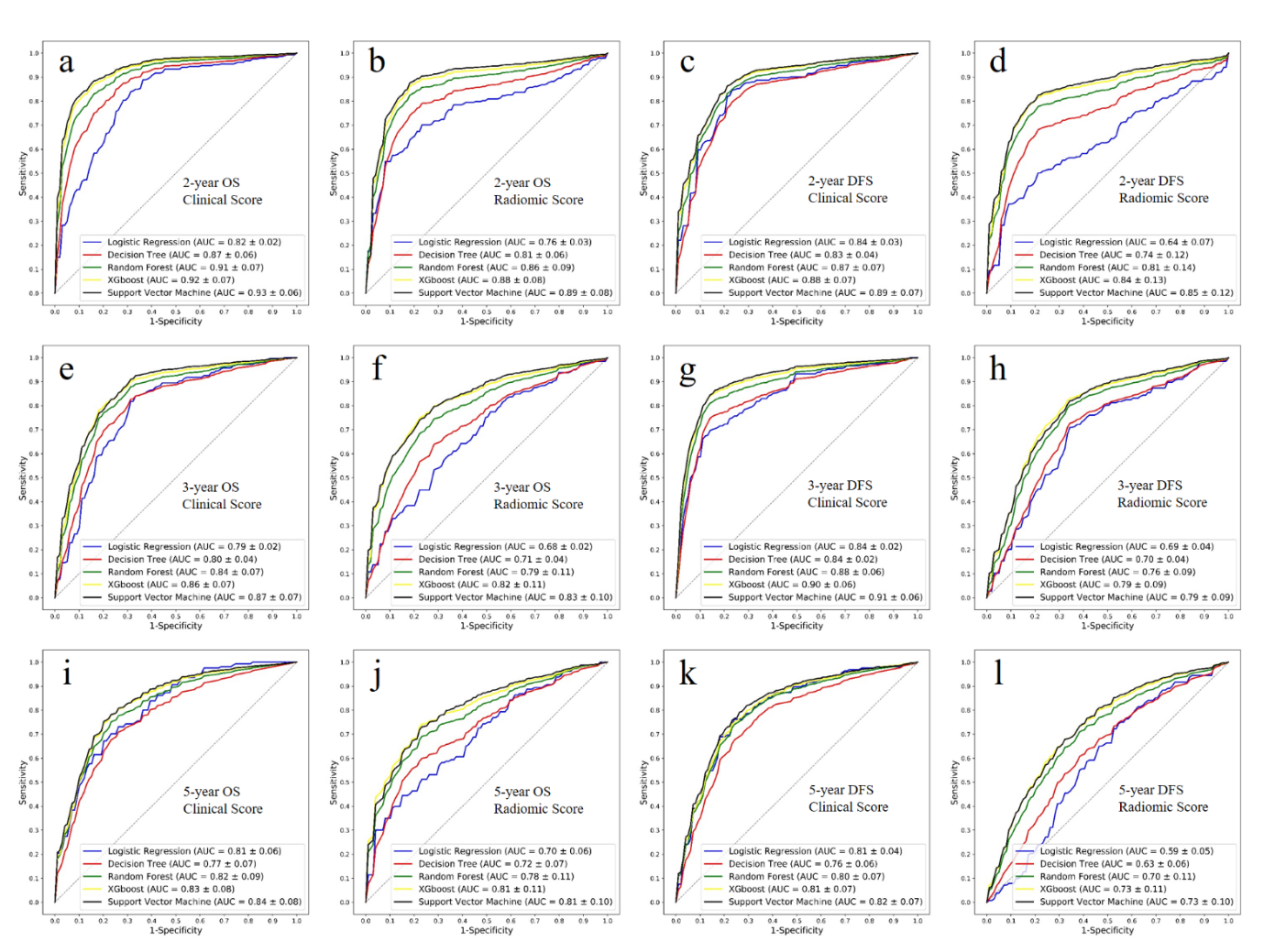


## **s-Figure 4. The ROC curves of clinical and radiomic scores in internal and external validation.**

OS, overall survival; DFS, disease free survival; ROC, receiver operating characteristic; AUC, area under the ROC curve.


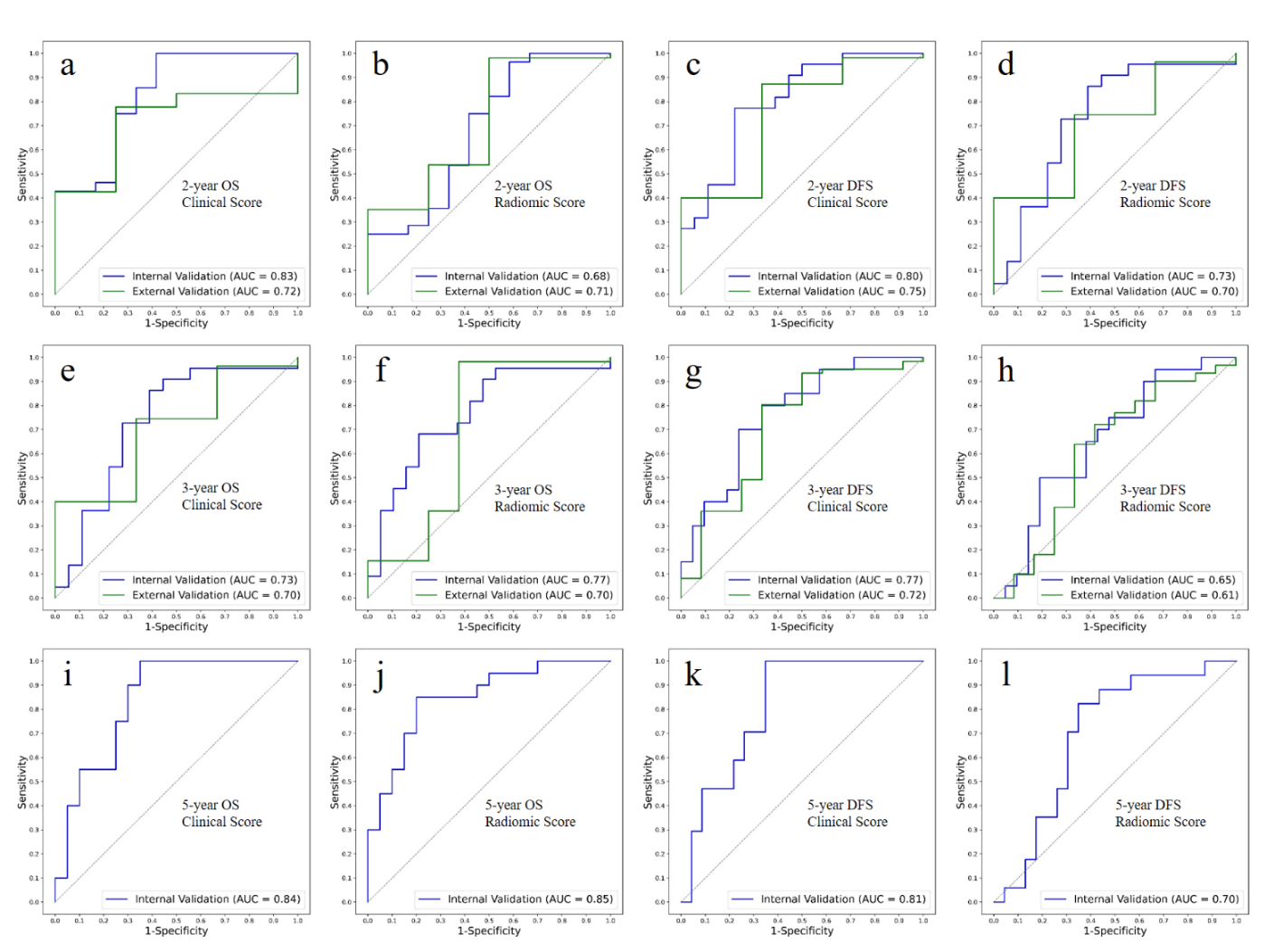


## **s-Figure 5. The combined nomograms.**

All the combined nomograms were built in the training cohort, and tested in the validation cohorts. OS, overall survival; DFS, disease free survival; CliScore, clinical score; RadScore, radiomics score.


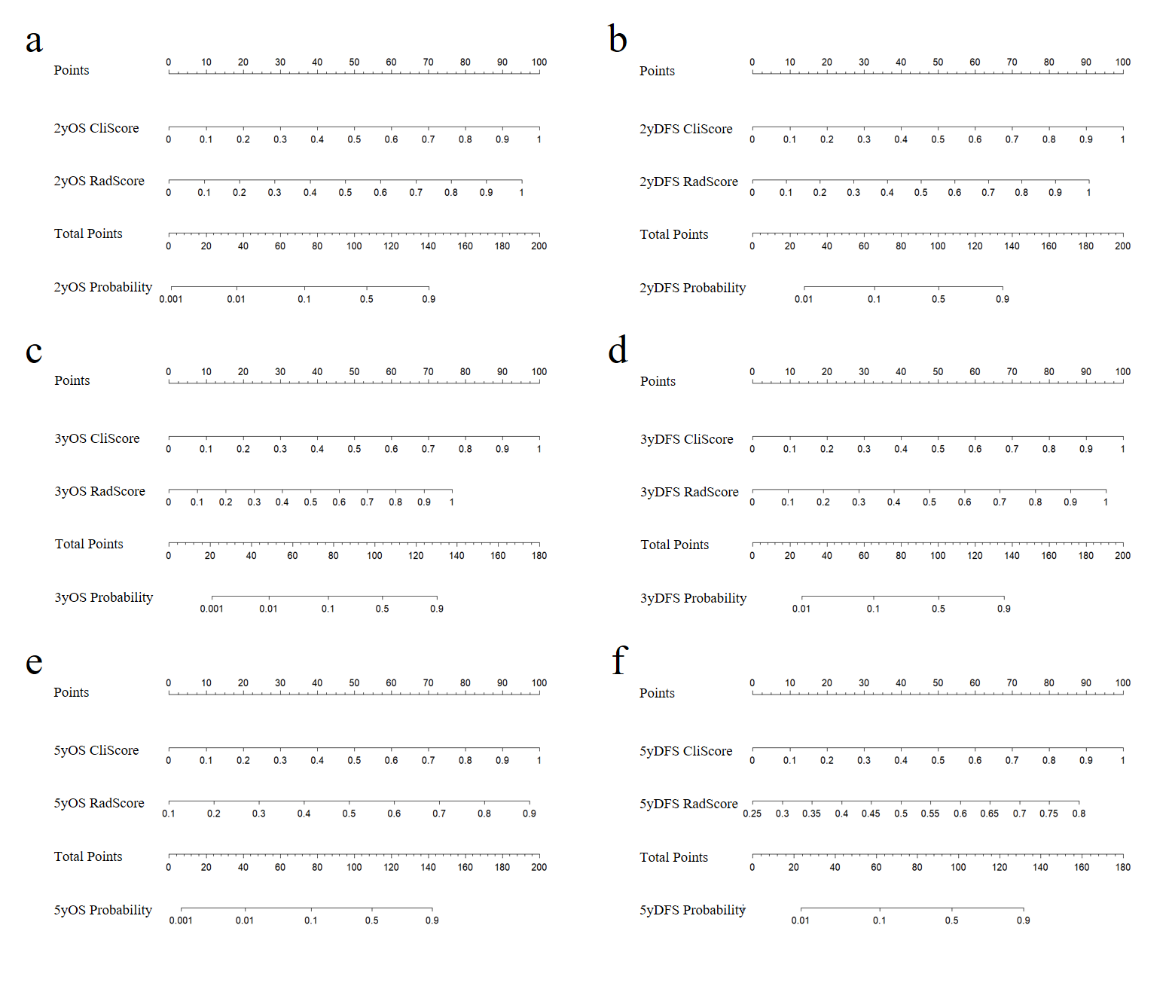


# **s-Tables**

## **s-Table 1. Literature review summary.**

OS, overall survival; DFS, disease-free survival; SEER, Surveillance, Epidemiology, and End Results; LNM, lymph nodes metastasis; AGR, albumin-to-globulin ratio; NLR, neutrophil-to-lymphocyte ratio; ER, estrogen receptor; PR, progesterone receptor; HER-2, human epidermal growth factor 2 receptor; PLR, platelet-to-lymphocyte ratio; WBC, white blood cells; BCSS, breast cancer-specific survival; AUC, area under curve; NAC, neoadjuvant chemotherapy; MRI, magnetic resonance imaging.

| Study | Period | Data source | No. of patients | Patient population | Statistical methods | Endpoint | Contributed clinical factors | Contributed pathological factors | Contributed experimental factors | Contributed radiology factors | Performance of models |
| --- | --- | --- | --- | --- | --- | --- | --- | --- | --- | --- | --- |
| Dai 2016[1] | 2004.1-2009.12 | Single Center | 247 | TNM stage I-IV | nomogram | OS | N stage | grade | CEA, CA153 | / | C-Index 0.664 (0.613–0.714) |
|  |  |  |  |  | nomogram | DFS | T stage, N stage | / | CEA, CA153 | / | C-Index 0.673 (0.626–0.720) |
| Dai 2018[2] | after 2010 | SEER | 6468 | TNM stage I-IV | nomogram | OS | age, race, laterality, location, size, stage, LNM, chemotherapy, radiotherapy | grade | / | / | C-Index 0.763 |
| Guo 2018[3] | 2010-2015 | SEER | 21419 | TNM stage I-IV | nomogram | OS | age, race, size, LNM, | histological type, grade | / | / | C-Index 0.774 (0.761–0.787) |
|  |  |  |  |  |  |  |  |  |  |  |  |
|  |  |  |  |  |  | BCSS | age, race, size, LNM, | histological type, grade | / | / | C-Index 0.792(0.778–0.806) |
| Lin 2018[4] | 2006.8-2014.7 | Single Center | 604 | TNM stage I-IV | nomogram | OS | family history, location, LNM | grade | CEA, CA153, CA125 | / | C-Index 0.76(0.72-0.81) |
|  |  |  |  |  |  |  |  |  |  |  |  |
| Cui 2019[5] | 2012.1-2016.2 | Single Center | 126 | TNM stage I-IV | nomogram | BCSS | LNM, TNM | grade | HIF-1a, c-myc | / | sensitivity 88.2% specificity 72.7% |
|  |  |  |  |  |  |  |  |  |  |  |  |
| Shi 2019[6] | 2008.3-2014.6 | Single Center | 379 | TNM stage I-IV | nomogram | OS | age, size, LNM | / | AGR, NLR | / | C-Index 0.74 (0.62–0.86) |
|  |  |  |  |  |  |  |  |  |  |  |  |
|  |  |  |  |  | nomogram | DFS | size, LNM | / | AGR, NLR | / | C-Index 0.69 (0.58–0.79) |
| Xu 2019[7] | 2000.1-2010.12 | Single Center | 1570 | TNM stage I-III | machine learning | OS | size, axillary surgery, breast surgery, chemotherapy | ER, PR, HER2, molecular subtypes | platelet, PLR, WBC, monocyte, neutrophile, NLR, lymphocyte | / | AUC 0.69-0.90 |
| Jiang 2020[8] | 2010.4-2017.4 | Single Center | 200 | TNM stage I-IV | radiomics nomogram | DFS | N stage | / | / | Mammography  Rad-score | C-Index 0.944 (0.883–1.004) |
|  |  |  |  |  |  |  |  |  |  |  |  |
| Wang 2020[9] | 2010-2016 | SEER | 1737 | with distant metastasis | nomogram | OS | age, marital status, T stage, bone, brain, liver and lung metastasis | / | / | / | C-Index 0.71-0.72 |
| Hua 2021[10] | 2005.9-2016.10 | Single Center | 358 | TNM stage I-II | nomogram | OS | T stage, N stage | / | Serum Iron Level | / | C-Index 0.739(0.666–0.812) |
|  |  |  |  |  |  |  |  |  |  |  |  |
|  |  |  |  |  |  | DFS | T stage, N stage | / | Serum Iron Level | / | C-Index 0.735(0.614–0.855) |
|  |  |  |  |  |  |  |  |  |  |  |  |
| Liu 2021[11] | 2010-2015 | SEER | 5677 | TNM stage I-IV | nomogram | OS | age, race, size, T stage, N stage, chemotherapy, radiotherapy, | grade | / | / | C-Index0.757(0.743–0.772) |
|  |  |  |  |  |  |  |  |  |  |  |  |
| Xia 2021[12] | 2011.8-2017.5 | Multicenter | 150 | after NAC | radiomics nomogram | DFS | number of lesions | pCR | / | MRI  Rad-score | C-Index 0.868(0.787–0.949) |
|  |  |  |  |  |  |  |  |  |  |  |  |
| Yu 2021[13] | 2012.7-2017.12 | Multicenter | 486 | TNM stage I-III | radiomics nomogram | DFS | size, LNM | ki67 expression | / | Ultrasound  Rad-score | C-Index 0.75(0.71–0.80) |
|  |  |  |  |  |  |  |  |  |  |  |  |
|  |  |  |  |  |  |  |  |  |  |  |  |
| Zhou 2021[14] | 2008.3-2019.2 | Single Center | 744 | TNM stage I-IV | nomogram | DFS | size, LNM | / | NLR, TILs | / | C-Index 0.717(0.665–0.769) |
|  |  |  |  |  |  |  |  |  |  |  |  |
|  |  |  |  |  |  | OS | age, size, LNM | / | NLR, TILs | / | C-Index 0.773 (0.719–0.827) |
|  |  |  |  |  |  |  |  |  |  |  |  |
| Zhu 2021[15] | 2015.2-2018.12 | Single Center | 165 | after NAC | nomogram | DFS | age, primary size, residual size | histological grade, lymphatic vessel invasion | / | / | C-Index 0.815 (0.779-0.851) |
|  |  |  |  |  |  |  |  |  |  |  |  |
| Huang 2022[16] | 2010-2016 | SEER | 4696 | TNM stage I-IV | machine learning | OS | / | / | / | / | AUC 0.837-0.882 |
|  |  |  |  |  |  | BCSS | / | / | / | / | AUC 0.879-0.908 |
| Ma 2022[17] | 2009.2-2018.12 | Single Center | 147 | after NAC | radiomics | recurrence | / | / | / | MRI radiomics model | AUC 0.933 (0.861–1) |
|  |  |  |  |  |  |  |  |  |  |  |  |
| Mao 2022[18] | 2010-2015 | SEER | 16997 | pT1-2N0M0 | nomogram | OS | age, size, laterality, mastectomy | / | / | / | C-Index 0.705 (0.688-0.721) |
|  |  |  |  |  |  |  |  |  |  |  |  |
| Sheng 2022[19] | 2011-2015 | Single Center | 636 | TNM stage I-IV | nomogram | DFS | LNM, chemotherapy | lymphatic vessel invasion | / | sonographic features | C-Index 0.694 AUC 0.488-0.787 |
|  |  |  |  |  |  |  |  |  |  |  |  |
|  |  |  |  |  |  |  |  |  |  |  |  |
| Wang 2022[20] | 2009.1-2018.6 | Single Center | 602 | after surgery | radiomics, machine learning, | DFS | size, number of LNM, stage | lymphatic vessel invasion | / | Ultrasound radiomics features | AUC 0.84-0.90 |
|  |  |  |  |  |  |  |  |  |  |  |  |
|  |  |  |  |  |  |  |  |  |  |  |  |
| Zhu 2022[21] | 2010-2015 | SEER | 998 | metaplastic after surgery | nomogram | OS | age, marital status, stage, surgery, chemotherapy, LNM | / | / | / | C-Index 0.73(0.71–0.75) AUC 0.735-0.785 |
|  |  |  |  |  |  |  |  |  |  |  |  |
|  |  |  |  |  |  |  |  |  |  |  |  |

## **s-Table 2 Patient characteristic of training, internal validation and external validation cohorts.**

| Characteristics | | Primary cohort (N=306) | Internal validation cohort (N=77) | External validation cohort (N=82) | P-value | Data source |
| --- | --- | --- | --- | --- | --- | --- |
| Age, median (range) (years) | | 49(28,81) | 45(26 ,81) | 46.5(26 ,81) | <0.01 | Electronic medical records |
| symptom, n (%) | yes | 56(18.30%) | 16(20.78%) | 22(26.83%) | 0.23 |  |
|  | no | 250(81.70%) | 61(79.22%) | 60(73.17%) |  |  |
| palpability, n (%) | yes | 277(90.52%) | 72(93.51%) | 75(91.46%) | 0.71 |  |
|  | no | 29(9.48%) | 5(6.49%) | 7(8.54%) |  |  |
| Pathological T staging, n (%) | 1 | 89(29.08%) | 19(24.68%) | 22(26.83%) | 0.93 |  |
|  | 2 | 185(60.46%) | 48(62.34%) | 48(58.54%) |  |  |
|  | 3 | 15(4.90%) | 4(5.19%) | 5(6.10%) |  |  |
|  | 4 | 17(5.56%) | 6(7.79%) | 7(8.54%) |  |  |
| Pathological N staging, n (%) | 0 | 166(54.25%) | 31(40.26%) | 36(43.90%) | 0.14 |  |
|  | 1 | 89(29.08%) | 27(35.06%) | 23(28.05%) |  |  |
|  | 2 | 24(7.84%) | 8(10.39%) | 9(10.98%) |  |  |
|  | 3 | 27(8.82%) | 11(14.29%) | 14(17.07%) |  |  |
| Pathological clinical staging, n (%) | 1 | 68(22.22%) | 12(15.58%) | 15(18.29%) | 0.43 |  |
|  | 2 | 172(56.21%) | 44(57.14%) | 43(52.44%) |  |  |
|  | 3 | 66(21.57%) | 21(27.27%) | 24(29.27%) |  |  |
| Surgical history, n (%) | yes | 27(8.82%) | 2(2.60%) | 4(4.88%) | 0.11 |  |
|  | no | 279(91.18%) | 75(97.40%) | 78(95.12%) |  |  |
| Gestation and production history, n (%) | yes | 301(98.37%) | 75(97.40%) | 78(95.12%) | 0.23 |  |
|  | no | 5(1.63%) | 2(2.60%) | 4(4.88%) |  |  |
| Surgery, n (%) | yes | 295(96.41%) | 73(94.81%) | 77(93.90%) | 0.56 |  |
|  | no | 11(3.59%) | 4(5.19%) | 5(6.10%) |  |  |
| Neoadjuvant chemotherapy, n (%) | yes | 44(14.38%) | 12(15.58%) | 17(20.73%) | 0.37 |  |
|  | no | 262(85.62%) | 65(84.42%) | 65(79.27%) |  |  |
| Treatment, n (%) | 1 | 251(82.03%) | 61(79.22%) | 60(73.17%) | 0.49 |  |
|  | 2 | 44(14.38%) | 12(15.58%) | 17(20.73%) |  |  |
|  | 3 | 11(3.59%) | 4(5.19%) | 5(6.10%) |  |  |
| Radiation therapy, n (%) | yes | 102(33.33%) | 32(41.56%) | 29(35.37%) | 0.40 |  |
|  | no | 204(66.67%) | 45(58.44%) | 53(64.63%) |  |  |
| Contralateral mastectomy, n (%) | yes | 11(3.59%) | 4(5.19%) | 2(2.44%) | 0.65 |  |
|  | no | 295(96.41%) | 73(94.81%) | 80(97.56%) |  |  |
| Breast-conserving surgery, n (%) | yes | 34(11.11%) | 8(10.39%) | 7(8.54%) | 0.80 |  |
|  | no | 272(88.89%) | 69(89.61%) | 75(91.46%) |  |  |
| Mixed pathological types, n (%) | yes | 104(33.99%) | 19(24.68%) | 22(26.83%) | 0.19 | BUPDIMS |
|  | no | 202(66.01%) | 58(75.32%) | 60(73.17%) |  |  |
| Histological grading, n (%) | 2 | 47(15.36%) | 11(14.29%) | 12(14.63%) | 0.83 |  |
|  | 3 | 259(84.64%) | 66(85.71%) | 70(85.37%) |  |  |
| HER-2 expression, n (%) | 0 | 150(49.02%) | 39(50.65%) | 51(62.20%) | 0.34 |  |
|  | 1 | 99(32.35%) | 24(31.17%) | 20(24.39%) |  |  |
|  | 2 | 57(18.63%) | 14(18.18%) | 11(13.41%) |  |  |
| Ki-67 expression, median (range) (%) | | 60(5,95) | 65(10 ,95) | 65(10 ,95) | 0.26 |  |
| Lymph node metastasis, n (%) | yes | 97(31.70%) | 27(35.06%) | 29(35.37%) | 0.75 |  |
|  | no | 209(68.30%) | 50(64.94%) | 53(64.63%) |  |  |
| Tumor size，median (range) (mm) | | 24(6,65) | 25(10 ,65) | 26(10 ,65) | 0.32 |  |
| Location, n (%) | left | 165(53.92%) | 36(46.75%) | 43(52.44%) | 0.53 |  |
|  | right | 141(46.08%) | 41(53.25%) | 39(47.56%) | 0.92 |  |
| Quadrant, n (%) | upper outer | 190(62.09%) | 44(57.14%) | 45(54.88%) |  |  |
|  | lower outer | 36(11.76%) | 11(14.29%) | 11(13.41%) |  |  |
|  | lower inner | 22(7.19%) | 6(7.79%) | 8(9.76%) |  |  |
|  | upper inner | 58(18.95%) | 16(20.78%) | 18(21.95%) |  |  |
| Number, n (%) | single nodules | 284(92.81%) | 68(88.31%) | 71(86.59%) | 0.15 |  |
|  | multiple nodules | 22(7.19%) | 9(11.69%) | 11(13.41%) |  |  |
| Orientation, n (%) | parallel | 161(52.61%) | 42(54.55%) | 41(50.00%) | 0.85 | Images re-review |
|  | non-parallel | 145(47.39%) | 35(45.45%) | 41(50.00%) | 0.85 |  |
| Margin, n (%) | circumscribed | 7(2.29%) | 8(10.39%) | 4(4.88%) | 0.45 |  |
|  | uncircumscribed | 299(97.71%) | 75(97.40%) | 78(95.12%) | 0.45 |  |
|  | indistinct | 289(94.44%) | 72(93.51%) | 75(91.46%) | 0.61 |  |
|  | angulate | 154(50.33%) | 36(46.75%) | 33(40.24%) | 0.26 |  |
|  | microlobular | 172(56.21%) | 37(48.05%) | 31(37.80%) | 0.01 |  |
|  | spiculate | 48(15.69%) | 10(12.99%) | 9(10.98%) | 0.52 |  |
| Echo pattern, n (%) | hypoechoic | 188(61.44%) | 50(64.94%) | 54(65.85%) | 0.67 |  |
|  | isoechoic | 39(12.75%) | 7(9.09%) | 6(7.32%) |  |  |
|  | complex | 79(25.82%) | 20(25.97%) | 22(26.83%) |  |  |
| Internal heterogeneous, n (%) | yes | 231(75.49%) | 52(67.53%) | 54(65.85%) | 0.13 |  |
|  | no | 75(24.51%) | 25(32.47%) | 28(34.15%) |  |  |
| Posterior features, n (%) | no | 138(45.10%) | 36(46.75%) | 44(53.66%) | 0.79 |  |
|  | enhancement | 132(43.14%) | 31(40.26%) | 29(35.37%) | 0.44 |  |
|  | shadowing | 29(9.48%) | 7(9.09%) | 6(7.32%) | 0.83 |  |
|  | combined | 7(2.29%) | 3(3.90%) | 3(3.66%) | 0.65 |  |
| Calcification, n (%) | no | 90(29.41%) | 33(42.86%) | 43(52.44%) | <0.01 |  |
|  | macro | 23(7.52%) | 4(5.19%) | 8(9.76%) | 0.55 |  |
|  | micro | 210(68.63%) | 42(54.55%) | 37(45.12%) | <0.01 |  |
| BI-RADS classification, n (%) | 4b | 29(9.48%) | 8(10.39%) | 9(10.98%) | 0.99 |  |
|  | 4c | 124(40.52%) | 30(38.96%) | 33(40.24%) |  |  |
|  | 5 | 153(50.00%) | 39(50.65%) | 40(48.78%) |  |  |
| Height，median (range) | | 232(59 ,505) | 250(94 ,488) | 240.5(83 ,488) | 0.16 | Computer-aided |
| Width，median (range) | | 342(107,763) | 364(136 ,746) | 355.5(136 ,763) | 0.54 |  |
| Height width ratio，median (range) | | 0.69(0.29 ,1.34) | 0.71(0.35 ,1.07) | 0.73(0.36,1.30) | 0.69 |  |
| Axillary lymph node, n (%) | yes | 161(52.61%) | 54(70.13%) | 52(63.41%) | 0.01 | BUPDIMS |
|  | no | 145(47.39%) | 23(29.87%) | 30(36.59%) |  |  |
| Clavicular lymph nodes, n (%) | yes | 43(14.05%) | 17(22.08%) | 19(23.17%) | 0.06 |  |
|  | no | 263(85.95%) | 60(77.92%) | 63(76.83%) |  |  |
| Ipsilateral breast abnormality, n (%) | yes | 98(32.03%) | 18(23.38%) | 26(31.71%) | 0.33 |  |
|  | no | 208(67.97%) | 59(76.62%) | 56(68.29%) |  |  |
| Contralateral breast abnormality, n (%) | yes | 118(38.56%) | 30(38.96%) | 33(40.24%) | 0.96 |  |
|  | no | 188(61.44%) | 47(61.04%) | 49(59.76%) |  |  |
| Contralateral lymph nodes abnormality, n (%) | yes | 20(6.54%) | 5(6.49%) | 6(7.32%) | 0.97 |  |
|  | no | 286(93.46%) | 72(93.51%) | 76(92.68%) |  |  |

## **s-Table 3** Valuable features across different radiomic and clinical scores.

| **Scores** | | **Features** |
| --- | --- | --- |
| **Clinical** | 2OS | age, h, tumor size, HWR, symptom, mixed pathological types, w, contralateral breast abnormality, treatment, pathological N staging |
|  | 2DFS | tumor size, age, w, HWR, pathological N staging, h, HER-2 expression, Ki-67 expression, BI-RADS classification, quadrant |
|  | 3OS | age, h, w, tumor size, HWR, pathological N staging, quadrant, mixed pathological types, HER-2 expression, pathological T staging |
|  | 3DFS | age, h, tumor size, HWR, symptom, contralateral breast abnormality, treatment, pathological N staging, quadrant |
|  | 5OS | age, tumor size, Ki-67 expression, w, h, HWR, pathological N staging, enhancement, quadrant, contralateral breast abnormality |
|  | 5DFS | w, tumor size, age, h, Ki-67 expression, HWR, pathological N staging, quadrant, clavicular lymph nodes, symptom |
| **Radiomics** | 2OS | firstorder_10Percentile, shape_Maximum2DDiameterRow, firstorder_Range, gldm_DependenceVariance, shape_SurfaceVolumeRatio, ngtdm_Coarseness, glrlm_RunLengthNonUniformityNormalized, glcm_Imc1, glcm_DifferenceVariance, firstorder_InterquartileRange |
|  | 2DFS | shape_Elongation, glcm_ClusterShade, firstorder_10Percentile, firstorder_Kurtosis, firstorder_90Percentile, glcm_Idm, shape_Maximum2DDiameterColumn, shape_Maximum2DDiameterSlice, glcm_Id, firstorder _Minimum |
|  | 3OS | glcm_DifferenceVariance, shape_Elongation, ngtdm_Coarseness, firstorder_10Percentile, gldm_Dependence Entropy, gldm_DependenceVariance, glcm_Idm, ngtdm_Strength, glcm_Imc2, glcm_Contrast |
|  | 3DFS | firstorder_10Percentile, ngtdm_Coarseness, gldm_DependenceVariance, shape_SurfaceVolumeRatio, shape_Elongation, glcm_Idm, firstorder_Mean, firstorder_Skewness, shape_Maximum2DDiameterRow, firstorder_Range |
|  | 5OS | firstorder_Energy, firstorder_Skewness, firstorder_Minimum, firstorder_10Percentile, glcm_Imc2, glcm_ ClusterShade, shape_SurfaceVolumeRatio, shape_Elongation, glcm_JointEnergy, glszm_ZoneVariance |
|  | 5DFS | firstorder_10Percentile, ngtdm_Coarseness, firstorder_Minimum, shape_SurfaceVolumeRatio, firstorder_ Kurtosis, shape_Maximum2DDiameterRow, shape_Elongation, glcm_MaximumProbability, ngtdm_Contrast, firstorder_Maximum |

## **s-Table 4. Comparison of clinicopathological and sonographic characteristics between progressed and non-progressed patients in two centers.**

| Characteristics | | Center 1 (N=383) | | |  | Center 2 (N=82) | | |
| --- | --- | --- | --- | --- | --- | --- | --- | --- |
|  |  | Progressed (N=56) | Non-progressed (N=327) | *p* |  | Progressed (N=11) | Non-progressed (N=71) | *p* |
| Age, median (range) (years) | | 50(28,81) | 48.18(26,81) | 0.060 |  | 57(45,76) | 45.13(26,81) | 0.002 |
| symptom, n (%) | yes | 12(21.43%) | 60(18.35%) | 0.586 |  | 3(27.27%) | 19(26.76%) | 0.972 |
|  | no | 44(78.57%) | 267(81.65%) |  |  | 8(72.73%) | 52(73.24%) |  |
| palpability, n (%) | yes | 45(80.36%) | 23(7.03%) | 0.002 |  | 8(72.73%) | 67(94.37%) | 0.017 |
|  | no | 11(19.64%) | 304(92.97%) |  |  | 3(27.27%) | 4(5.63%) |  |
| Pathological T staging, n (%) | 1 | 13(23.21%) | 95(29.05%) | 0.423 |  | 4(36.36%) | 18(25.35%) | 0.803 |
|  | 2 | 34(60.71%) | 199(60.86%) |  |  | 5(45.45%) | 43(60.56%) |  |
|  | 3 | 5(8.93%) | 14(4.28%) |  |  | 1(9.09%) | 4(5.63%) |  |
|  | 4 | 4(7.14%) | 19(5.81%) |  |  | 1(9.09%) | 6(8.45%) |  |
| Pathological N staging, n (%) | 0 | 22(39.29%) | 175(53.52%) | 0.150 |  | 5(45.45%) | 31(43.66%) | 0.781 |
|  | 1 | 20(35.71%) | 96(29.36%) |  |  | 3(27.27%) | 20(28.17%) |  |
|  | 2 | 8(14.29%) | 24(7.34%) |  |  | 2(18.18%) | 7(9.86%) |  |
|  | 3 | 6(10.71%) | 32(9.79%) |  |  | 1(9.09%) | 13(18.31%) |  |
| Clinical staging, n (%) | 1 | 9(16.07%) | 71(21.71%) | 0.041 |  | 3(27.27%) | 12(16.90%) | 0.706 |
|  | 2 | 27(48.21%) | 189(57.80%) |  |  | 5(45.45%) | 38(53.52%) |  |
|  | 3 | 20(35.71%) | 67(20.49%) |  |  | 3(27.27%) | 21(29.58%) |  |
| Surgical history, n (%) | yes | 3(5.36%) | 26(7.95%) | 0.784 |  | 2(18.18%) | 2(2.82%) | 0.028 |
|  | no | 53(94.64%) | 301(92.05%) |  |  | 9(81.82%) | 69(97.18%) |  |
| Gestation and production history, n (%) | yes | 55(98.21%) | 321(98.17%) | 1.000 |  | 11(100.00%) | 67(94.37%) | 1.000 |
|  | no | 1(1.79%) | 6(1.83%) |  |  | 0(0.00%) | 4(5.63%) |  |
| Surgery, n (%) | yes | 54(96.43%) | 314(96.02%) | 1.000 |  | 10(90.91%) | 67(94.37%) | 0.523 |
|  | no | 2(3.57%) | 13(3.98%) |  |  | 1(9.09%) | 4(5.63%) |  |
| Neoadjuvant chemotherapy, n (%) | yes | 14(25.00%) | 42(12.84%) | 0.017 |  | 1(9.09%) | 16(22.54%) | 0.306 |
|  | no | 42(75.00%) | 285(87.16%) |  |  | 10(90.91%) | 55(77.46%) |  |
| Treatment, n (%) | 1 | 40(71.43%) | 272(83.18%) | 0.059 |  | 9(81.82%) | 51(71.83%) | 0.564 |
|  | 2 | 14(25.00%) | 42(12.84%) |  |  | 1(9.09%) | 16(22.54%) |  |
|  | 3 | 2(3.57%) | 13(3.98%) |  |  | 1(9.09%) | 4(5.63%) |  |
| Radiation therapy, n (%) | yes | 20(35.71%) | 114(34.86%) | 0.902 |  | 4(36.36%) | 25(35.21%) | 0.941 |
|  | no | 36(64.29%) | 213(65.14%) |  |  | 7(63.64%) | 46(64.79%) |  |
| Contralateral mastectomy, n (%) | yes | 3(5.36%) | 12(3.67%) | 0.468 |  | 0(0.00%) | 2(2.82%) | 1.000 |
|  | no | 53(94.64%) | 315(96.33%) |  |  | 11(100.00%) | 69(97.18%) |  |
| Breast-conserving surgery, n (%) | yes | 3(5.36%) | 39(11.93%) | 0.171 |  | 0(0.00%) | 7(9.86%) | 0.586 |
|  | no | 53(94.64%) | 288(88.07%) |  |  | 11(100.00%) | 64(90.14%) |  |
| Mixed pathological types, n (%) | yes | 12(21.43%) | 111(33.94%) | 0.064 |  | 3(27.27%) | 19(26.76%) | 1.000 |
|  | no | 44(78.57%) | 216(66.05%) |  |  | 8(72.73%) | 52(73.24%) |  |
| Histological grading, n (%) | 2 | 9(16.07%) | 49(14.98%) | 0.834 |  | 2(18.18%) | 10(14.08%) | 0.721 |
|  | 3 | 47(83.93%) | 278(85.02%) |  |  | 9(81.82%) | 61(85.92%) |  |
| HER-2 expression, n (%) | 0 | 27(48.21%) | 162(49.54%) | 0.780 |  | 7(63.64%) | 44(61.97%) | 0.218 |
|  | 1 | 20(35.71%) | 103(31.50%) |  |  | 1(9.09%) | 19(26.76%) |  |
|  | 2 | 9(16.07%) | 62(18.96%) |  |  | 3(27.27%) | 8(11.27%) |  |
| Ki-67 expression, median (range) (%) | | 57.5(5,90) | 59.26(5,95) | 0.298 |  | 64(50,85) | 64(10,95) | 0.262 |
| Lymph node metastasis, n (%) | yes | 19(33.93%) | 105(32.11%) | 0.788 |  | 4(36.36%) | 25(35.21%) | 0.941 |
|  | no | 37(66.07%) | 222(67.89%) |  |  | 7(63.64%) | 46(64.79%) |  |
| Tumor size，median (range) (mm) | | 25(9,62) | 24.04(6,65) | 0.548 |  | 25(12,52) | 26.25(10,65) | 0.395 |
| Location, n (%) | left | 29(51.79%) | 172(52.60%) | 0.910 |  | 5(45.45%) | 38(53.52%) | 0.618 |
|  | right | 27(48.21%) | 155(47.40%) |  |  | 6(54.55%) | 33(46.48%) |  |
| Quadrant, n (%) | upper outer | 42(75.00%) | 192(58.72%) | 0.089 |  | 7(63.64%) | 38(53.52%) | 0.409 |
|  | lower outer | 6(10.71%) | 41(12.54%) |  |  | 0(0.00%) | 11(15.49%) |  |
|  | lower inner | 1(1.79%) | 27(8.26%) |  |  | 2(18.18%) | 6(8.45%) |  |
|  | upper inner | 7(12.50%) | 67(20.49%) |  |  | 2(18.18%) | 16(22.54%) |  |
| Number, n (%) | single nodules | 49(87.50%) | 303(92.66%) | 0.191 |  | 11(100.00%) | 60(84.51%) | 0.345 |
|  | multiple nodules | 7(12.50%) | 24(7.34%) |  |  | 0(0.00%) | 11(15.49%) |  |
| Orientation, n (%) | parallel | 29(51.79%) | 153(46.79%) | 0.843 |  | 6(54.55%) | 35(49.30%) | 0.746 |
|  | non-parallel | 27(48.21%) | 174(53.21%) |  |  | 5(45.45%) | 36(50.70%) |  |
| Margin, n (%) | circumscribed | 0(0.00%) | 9(2.75%) | 0.368 |  | 1(9.09%) | 3(4.23%) | 0.444 |
|  | uncircumscribed | 56(100.00%) | 318(97.25%) |  |  | 10(90.91%) | 68(95.77%) |  |
|  | indistinct | 54(96.43%) | 307(93.88%) | 0.450 |  | 9(81.82%) | 66(92.96%) | 0.236 |
|  | angulate | 34(60.71%) | 156(47.71%) | 0.072 |  | 4(36.36%) | 29(40.85%) | 1.000 |
|  | microlobular | 33(58.93%) | 176(53.82%) | 0.478 |  | 4(36.36%) | 27(38.03%) | 1.000 |
|  | spiculate | 15(26.79%) | 43(13.15%) | 0.009 |  | 0(0.00%) | 9(12.68%) | 0.601 |
| Echo pattern, n (%) | hypoechoic | 34(60.71%) | 204(62.39%) | 0.972 |  | 7(63.64%) | 47(66.20%) | 0.968 |
|  | isoechoic | 7(12.50%) | 39(11.93%) |  |  | 1(9.09%) | 5(7.04%) |  |
|  | complex | 15(26.79%) | 84(25.69%) |  |  | 3(27.27%) | 19(26.76%) |  |
| Internal heterogeneous, n (%) | yes | 37(66.07%) | 246(75.23%) | 0.149 |  | 5(45.45%) | 22(30.99%) | 0.173 |
|  | no | 19(33.93%) | 81(24.77%) |  |  | 6(54.55%) | 49(69.01%) |  |
| Posterior features, n (%) | no | 25(44.64%) | 149(45.57%) | 0.254 |  | 5(45.45%) | 39(54.93%) | 0.411 |
|  | enhancement | 20(35.71%) | 143(43.73%) |  |  | 6(54.55%) | 23(32.39%) |  |
|  | shadowing | 9(16.07%) | 27(8.26%) |  |  | 0(0.00%) | 6(8.45%) |  |
|  | combined | 2(3.57%) | 8(2.45%) |  |  | 0(0.00%) | 3(4.23%) |  |
| Calcification, n (%) | no | 22(39.29%) | 101(30.89%) | 0.214 |  | 7(63.64%) | 36(50.70%) | 0.424 |
|  | macro | 7(12.50%) | 20(6.12%) | 0.085 |  | 0(0.00%) | 8(11.27%) | 0.590 |
|  | micro | 31(55.36%) | 221(67.58%) | 0.075 |  | 4(36.36%) | 33(46.48%) | 0.746 |
| BI-RADS classification, n (%) | 4b | 3(5.36%) | 34(10.40%) | 0.380 |  | 4(36.36%) | 5(7.04%) | 0.015 |
|  | 4c | 26(46.43%) | 128(39.14%) |  |  | 3(27.27%) | 30(42.25%) |  |
|  | 5 | 27(48.21%) | 165(50.46%) |  |  | 4(36.36%) | 36(50.70%) |  |
| Height，median (range) | | 245.50(79.462) | 235.13(59,505) | 0.709 |  | 210(83,339) | 252.33(94,488) | 0.026 |
| Width，median (range) | | 325.50(107,756) | 350.67(109,763) | 0.544 |  | 291(170,417) | 361(136,763) | 0.161 |
| Height width ratio，median (range) | | 0.73(0.35,1.34) | 0.69(0.29,1.30) | 0.179 |  | 0.61(0.49,0.87) | 0.76(0.36,1.30) | 0.105 |
| Axillary lymph node, n (%) | yes | 37(66.07%) | 149(45.57%) | 0.105 |  | 3(27.27%) | 49(69.01%) | 0.015 |
|  | no | 19(33.93%) | 178(54.43%) |  |  | 8(72.73%) | 22(30.99%) |  |
| Clavicular lymph nodes, n (%) | yes | 12(21.43%) | 48(14.68%) | 0.199 |  | 3(27.27%) | 16(22.54%) | 0.711 |
|  | no | 44(78.57%) | 279(85.32%) |  |  | 8(72.73%) | 55(77.46%) |  |
| Ipsilateral breast abnormality, n (%) | yes | 13(23.21%) | 103(31.50%) | 0.271 |  | 2(18.18%) | 24(33.80%) | 0.489 |
|  | no | 43(76.79%) | 224(68.50%) |  |  | 9(81.82%) | 47(66.20%) |  |
| Contralateral breast abnormality, n (%) | yes | 16(28.57%) | 132(40.37%) | 0.094 |  | 1(9.09%) | 32(45.07%) | 0.043 |
|  | no | 40(71.43%) | 195(59.63%) |  |  | 10(90.91%) | 39(54.93%) |  |
| Contralateral lymph nodes abnormality, n (%) | yes | 3(5.36%) | 22(6.73%) | 1.000 |  | 0(0.00%) | 6(8.45%) | 1.000 |
|  | no | 53(94.64%) | 305(93.27%) |  |  | 11(100.00%) | 65(91.55%) |  |

## **s-Table 5 Survival analysis of** prediction probability of clinical score, radiomic score and combined nomograms.

| Models | | | Χ^2^ | *p* value |
| --- | --- | --- | --- | --- |
| Overall survival | 2-year | Clinical | 753.199 | <0.001 |
|  |  | Radiomic | 1487.859 | <0.001 |
|  |  | Combined | 1487.859 | <0.001 |
|  | 3-year | Clinical | 796.88 | <0.001 |
|  |  | Radiomic | 1487.075 | <0.001 |
|  |  | Combined | 1487.859 | <0.001 |
|  | 5-year | Clinical | 809.284 | <0.001 |
|  |  | Radiomic | 1708.356 | <0.001 |
|  |  | Combined | 1708.450 | <0.001 |
| Disease-free survival | 2-year | Clinical | 353.658 | <0.001 |
|  |  | Radiomic | 1521.019 | <0.001 |
|  |  | Combined | 1521.019 | <0.001 |
|  | 3-year | Clinical | 387.805 | <0.001 |
|  |  | Radiomic | 1520.998 | <0.001 |
|  |  | Combined | 1521.019 | <0.001 |
|  | 5-year | Clinical | 679.188 | <0.001 |
|  |  | Radiomic | 1647.212 | <0.001 |
|  |  | Combined | 1647.314 | <0.001 |

## **s-Table 6. RQS^[22]^ analysis of the study.** RQS, radiomics quality score.

| Criteria | Points | RQS |
| --- | --- | --- |
| Image protocol quality | + 1 (if protocols are well-documented) + 1 (if public protocol is used) | + 2 |
| Multiple segmentations | + 1 | + 1 |
| Phantom study on all scanners | + 1 | + 0 |
| Imaging at multiple time points | + 1 | + 0 |
| Feature reduction or adjustment for multiple testing | - 3 (if neither measure is implemented) + 3 (if either measure is implemented) | + 3 |
| Multivariable analysis with non-radiomics features | + 1 | + 1 |
| Detect and discuss biological correlates | + 1 | + 0 |
| Cut-off analyses | + 1 | + 1 |
| Discrimination statistics | + 1 (if a discrimination statistic and its statistical significance are reported) + 1 (if a resampling method technique is also applied) | + 2 |
| Calibration statistics | + 1 (if a calibration statistic and its statistical significance are reported) + 1 (if a resampling method technique is also applied) | + 2 |
| Prospective study registered in a trial database | + 7 (for prospective validation of a radiomics signature in an appropriate trial) | + 7 |
| Validation | - 5 (if validation is missing) + 2 (if validation is based on a dataset from the same institute) + 3 (if validation is based on a dataset from another institute) + 4 (if validation is based on two datasets from two  distinct institutes) + 4 (if the study validates a previously published  signature) + 5 (if validation is based on three or more datasets from distinct institutes) | + 4 |
| Comparison to ‘gold standard’ | + 2 | + 2 |
| Potential clinical utility | + 2 | + 2 |
| Cost-effectiveness analysis | + 1 | + 0 |
| Open science and data | + 1 (if scans are open source) + 1 (if region of interest segmentations are open source) + 1 (if code is open source) + 1 (if radiomics features are calculated on a set of representative ROIs and the calculated features and representative ROIs are open source) | + 3 |
| Total points = 36 | | 30 (83.3%) |

# Reference

1. Dai D, Chen B, Tang H, Wang B, Zhao Z, Xie X, et al. Nomograms for Predicting the Prognostic Value of Pre-Therapeutic CA15-3 and CEA Serum Levels in TNBC Patients. Plos One. 2016;11(8).

2. Dai D, Jin H, Wang X. Nomogram for predicting survival in triple-negative breast cancer patients with histology of infiltrating duct carcinoma: a population-based study. American Journal of Cancer Research. 2018;8(8):1576-85.

3. Guo L-W, Jiang L-M, Gong Y, Zhang H-H, Li X-G, He M, et al. Development and validation of nomograms for predicting overall and breast cancer-specific survival among patients with triple-negative breast cancer. Cancer Management and Research. 2018;10:5881-94.

4. Lin Y, Fu F, Lin S, Qiu W, Zhou W, Lv J, et al. A nomogram prediction for the survival of patients with triple negative breast cancer. Oncotarget. 2018;9(63):32108-18.

5. Cui J, Jiang H. Prediction of postoperative survival of triple-negative breast cancer based on nomogram model combined with expression of HIF-1 alpha and c-myc. Medicine. 2019;98(40).

6. Shi H, Wang X-H, Gu J-W, Guo G-L. Development and Validation of Nomograms for Predicting the Prognosis of Triple-Negative Breast Cancer Patients Based on 379 Chinese Patients. Cancer Management and Research. 2019;11:10827-39.

7. Xu Y, Ju L, Tong J, Zhou C, Yang J. Supervised Machine Learning Predictive Analytics For Triple-Negative Breast Cancer Death Outcomes. Oncotargets and Therapy. 2019;12:9059-67.

8. Jiang X, Zou X, Sun J, Zheng A, Su C. A Nomogram Based on Radiomics with Mammography Texture Analysis for the Prognostic Prediction in Patients with Triple-Negative Breast Cancer. Contrast Media & Molecular Imaging. 2020;2020.

9. Wang Z, Wang H, Sun X, Fang Y, Lu S-S, Ding S-N, et al. A Risk Stratification Model for Predicting Overall Survival and Surgical Benefit in Triple-Negative Breast Cancer Patients With de novo Distant Metastasis. Frontiers in Oncology. 2020;10.

10. Hua X, Duan F, Huang J, Bi X, Xia W, Song C, et al. A Novel Prognostic Model Based on the Serum Iron Level for Patients With Early-Stage Triple-Negative Breast Cancer. Frontiers in cell and developmental biology. 2021;9:777215-.

11. Liu X, Yue S, Huang H, Duan M, Zhao B, Liu J, et al. Risk Stratification Model for Predicting the Overall Survival of Elderly Triple-Negative Breast Cancer Patients: A Population-Based Study. Frontiers in Medicine. 2021;8.

12. Xia BQ, Wang H, Wang Z, Qian ZX, Xiao Q, Liu Y, et al. A Combined Nomogram Model to Predict Disease-free Survival in Triple-Negative Breast Cancer Patients With Neoadjuvant Chemotherapy. Frontiers in Genetics. 2021;12.

13. Yu F, Hang J, Deng J, Yang B, Wang J, Ye X, et al. Radiomics features on ultrasound imaging for the prediction of disease-free survival in triple negative breast cancer: a multi-institutional study. British Journal of Radiology. 2021;94(1126).

14. Zhou J-Y, Lu K-K, Fu W-D, Shi H, Gu J-W, Lu Y-Q, et al. Development of prognostic nomograms using institutional data for patients with triple-negative breast cancer. Future Oncology. 2021;17(36):5077-91.

15. Zhu M, Liang C, Zhang F, Zhu L, Chen D. A Nomogram to Predict Disease-Free Survival Following Neoadjuvant Chemotherapy for Triple Negative Breast Cancer. Frontiers in Oncology. 2021;11.

16. Huang K, Zhang J, Yu Y, Lin Y, Song C. The impact of chemotherapy and survival prediction by machine learning in early Elderly Triple Negative Breast Cancer (eTNBC): a population based study from the SEER database. Bmc Geriatrics. 2022;22(1).

17. Ma M, Gan L, Liu Y, Jiang Y, Xin L, Liu Y, et al. Radiomics features based on automatic segmented MRI images: Prognostic biomarkers for triple-negative breast cancer treated with neoadjuvant chemotherapy. European Journal of Radiology. 2022;146.

18. Mao Q, Liu S, Lv M, Sun Y, Zhang C, Li L. Nomogram for Predicting Overall Survival and Assessing the Survival Benefit of Adjuvant Treatment in pT1-2N0M0 Triple-Negative Breast Cancer: A Surveillance, Epidemiology, and End Results-Based Study. Frontiers in Oncology. 2022;11.

19. Sheng D-l, Shen X-g, Shi Z-t, Chang C, Li J-w. Survival outcome assessment for triple-negative breast cancer: a nomogram analysis based on integrated clinicopathological, sonographic, and mammographic characteristics. European Radiology. 2022.

20. Wang H, Li X, Yuan Y, Tong Y, Zhu S, Huang R, et al. Association of machine learning ultrasound radiomics and disease outcome in triple negative breast cancer. American Journal of Cancer Research. 2022;12(1):152-+.

21. Zhu K, Chen Y, Guo R, Dai L, Wang J, Tang Y, et al. Prognostic Factor Analysis and Model Construction of Triple-Negative Metaplastic Breast Carcinoma After Surgery. Frontiers in Oncology. 2022;12.

22. Lambin P, Leijenaar RTH, Deist TM, Peerlings J, de Jong EEC, van Timmeren J, et al. Radiomics: the bridge between medical imaging and personalized medicine. Nature reviews Clinical oncology. 2017;14(12):749-62.
